# TAK1 activation of alpha-TAT1 and microtubule hyperacetylation control AKT signaling and cell growth

**DOI:** 10.1038/s41467-018-04121-y

**Published:** 2018-04-27

**Authors:** Nirav Shah, Sanjay Kumar, Naveed Zaman, Christopher C. Pan, Jeffrey C. Bloodworth, Wei Lei, John M. Streicher, Nadine Hempel, Karthikeyan Mythreye, Nam Y. Lee

**Affiliations:** 1 Division of Pharmacology, College of Pharmacy, The Ohio State University, Columbus, OH USA; 2 Deparment of Pharmacology, College of Medicine, University of Arizona, Tucson, AZ USA; 3 Department of Pharmacology and Cancer Biology, Duke University, Durham, NC USA; 4 Department of Biochemistry and Molecular Biology, Loyola University Chicago, Chicago, IL USA; 5 Department of Pharmacology, Penn State University, State College, PA USA; 6 Department of Chemistry and Biochemistry, University of South Carolina, Columbia, SC USA; 7 Department of Chemistry and Biochemistry, University of Arizona, Tucson, AZ USA; 8 The University of Arizona Cancer Center, Tucson, AZ USA

## Abstract

Acetylation of microtubules (MT) confers mechanical stability necessary for numerous functions including cell cycle and intracellular transport. Although αTAT1 is a major MT acetyltransferase, how this enzyme is regulated remains much less clear. Here we report TGF-β-activated kinase 1 (TAK1) as a key activator of αTAT1. TAK1 directly interacts with and phosphorylates αTAT1 at Ser237 to critically enhance its catalytic activity, as mutating this site to alanine abrogates, whereas a phosphomimetic induces MT hyperacetylation across cell types. Using a custom phospho-αTAT1-Ser237 antibody, we screen various mouse tissues to discover that brain contains some of the highest TAK1-dependent αTAT1 activity, which, accordingly, is diminished rapidly upon intra-cerebral injection of a TAK1 inhibitor. Lastly, we show that TAK1 selectively inhibits AKT to suppress mitogenic and metabolism-related pathways through MT-based mechanisms in culture and in vivo. Collectively, our findings support a fundamental new role for TGF-β signaling in MT-related functions and disease.

## Abstract (web summary)

Acetylation of microtubules (MT) confers mechanical stability necessary for numerous cellular functions but its regulation is unclear. Here the authors show that the MT acetyltransferase αTAT1 is regulated by TGF-β-activated kinase 1 implicating TGF-β signaling in MT-related functions and disease.

## Introduction

Microtubules (MTs) are a fundamental part of the eukaryotic cytoskeletal system that coordinates diverse cellular processes including cell cycle, cell polarity, and intracellular transport^1–3^. As highly dynamic structures comprised α and β tubulin heterodimers, MTs continuously polymerize and depolymerize through interactions with various motor proteins and MT-associated proteins (MAPs). In addition, numerous posttranslational modifications (PTMs) have been shown to modulate its overall stability and function^4,5^. Among them, acetylation of α-tubulin at Lys40 is particularly intriguing, as it is the only PTM known to occur on the luminal side yet exerts distinct effects on intracellular transport of vesicles and protein cargo occurring on the outer surface of MTs.

Among the known acetyltransferases, there is mounting evidence that α-tubulin acetyltransferase 1 (αTAT1), a mammalian ortholog of MEC17 found in *Caenorhabditis elegans*, is considered the major, if not sole, MT-acetylating enzyme in vivo. Indeed, two independently derived αTAT1 knockout mouse models confirm that virtually all cell and tissue types are devoid of α-tubulin acetylation^6,7^. Surprisingly, αTAT1 is not essential for survival during development, although sensory deficits and other higher cognitive functions are likely compromised^8^. Its functional roles are more evident at the cellular level where αTAT1 dysfunction underlies defects in ciliary mechanotransduction, contact inhibition of proliferation, and directional cell migration^9,10^. αTAT1 may also have critical pathophysiologic roles in cancer and many neurodegenerative conditions^7,10,11^. These findings together with previous biophysical structural characterization provide clear fundamental roles for αTAT1 in MT functional modifications. However, aside from a study linking reactive oxygen species (ROS) and AMPK to MT hyperacetylation, there has been very limited knowledge of how this enzyme is catalytically regulated^12^.

Transforming growth factor β (TGF-β) is a multifunctional cytokine belonging to a superfamily of more than 30 members^13,14^. The prototype TGF-β1 along with many other members signal through the canonical type I receptor TβRI (ALK5) to elicit small mothers against decapentaplegic (SMAD) 2/3 transcriptional responses^15,16^. TGF-β activation of the SMAD-independent pathways occurs primarily through TGF-β-activated kinase 1 (TAK1), a serine/threonine kinase with essential roles in viability and homeostasis in a variety of cell types and organs^17–19^. TAK1 was initially identified as an effector of TGF-β-induced p38/JNK activation, although bone morphogenetic proteins, pro-inflammatory molecules such as TNFα and lipopolysaccharides (LPSs), and many others have now been identified as inducers^20–22^. Upon TGF-β stimulation, TAK1 phosphorylates the IKK complex and mitogen-activated protein kinase kinases to induce nuclear factor-κB and mitogen-activated protein kinases such as ERK, p38, and JNK^23^.

Although MTs mediate the intracellular transport of numerous signaling molecules including SMADs, it is currently unknown whether any of these TGF-β effectors directly impact MT functions in real time. This is in stark contrast to a number of studies demonstrating rapid, SMAD-independent mechanisms by which TGF-β controls actin cytoskeletal remodeling^24,25^. In the present study, we establish a fundamental new role for TGF-β signaling in MT dynamics and function. Through mass spectrometry and proteomics screening, we identified αTAT1 as a novel binding partner for TAK1. Here we describe the mechanisms by which TGF-β exerts rapid, non-transcriptional effects on MT acetylation and downstream cellular functions through TAK1.

## Results

### TAK1 directly binds to αTAT1 to enhance MT acetylation

**Fig. 1:**
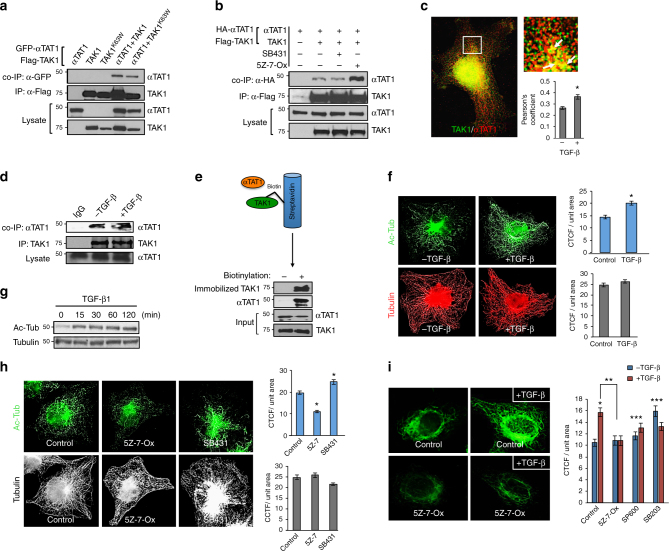
**TAK1 directly binds αTAT1 to promote MT acetylation. a** COS-7 cells expressing HA-αTAT1 WT, Flag-TAK1 WT, or Flag-TAK1 kinase-dead (K63W) were immunoprecipitated with Flag antibody. **b** COS-7 cells expressing HA-αTAT1 WT with or without Flag-TAK1 WT were treated with SB431542 (30 μM) or (5Z)-7-Oxozeaenol (20 μM) for 30 min before immunoprecipitation with Flag antibody. **c** COS-7 cells were treated with TGF-β1 (200 pM) for 30 min before fixing and then imaged for endogenous αTAT1 (C6ORF) and TAK1 immunofluorescence staining. At least 15 cells per condition (3 ROIs per cell) were quantified using Image J plugin JACoP to determine Pearson’s correlation coefficient. Error bars represent SEM from three independent experiments (*p = 0.0001). **d** COS-7 cells were treated with TGF-β1 (200 pM) for 30 min and the lysates were immunoprecipitated with either IgG control or αTAT1 (C6ORF) antibody. **e** Purified αTAT1 and TAK1 were incubated with biotinylated TAK1 immobilized to streptavidin agarose. As a negative control, αTAT1 and non-biotinylated TAK1 were incubated in streptavidin agarose. **f** COS-7 cells were treated with TGF-β1 (200 pM) for 30 min before fixation, then imaged for acetyl-tubulin (top) and total tubulin (bottom) immunofluorescence staining. The images were quantified for corrected total cell fluorescence (CTCF) per unit area using Image J. Quantification was based on data collected from 50 cells per condition. The images are representative of three independent experiments, *n* = 3. Error bars represent SEM and type 2 *t*-test results show relative to control: *p < 0.05. **g** Western blot analysis of acetyl and total tubulin post TGF-β1 (200 pM) treatment for 0, 15, 30, 60, and 120 min in COS-7 cells. **h** COS-7 cells were treated with (5Z)-7-Oxozeaenol (20 μM) or SB431542 (30 μM) for 30 min before fixation, then imaged for Acetyl-tubulin (top) and total tubulin (bottom three) immunofluorescence staining. At least 50 cells per condition were quantified for CTCF using Image J. The images are representative of three independent experiments, *n* = 3. Error bars represent SEM and type 2 *t*-test results show relative to control: *p < 0.05. **i** HeLa cells were pre-treated with (5Z)-7-Oxozeaenol (20 μM), SP600125 (10 μM), or SB203580 (20 μM) for 30 min and then subjected to TGF-β1 (200 pM) for 30 min before fixation. The images represent Acetyl-tubulin immunofluorescence staining and were quantified for CTCF using Image J. Quantification was based on data collected from at least 30 cells per condition. The images are representative of three independent experiments, *n* = 3. Error bars represent SEM. Relative to control: *p < 0.05; **p < 0.05; ***NS

In search of new binding partners for the major TGF-β effectors present in most cell types, we performed a mass spectrometry-based proteomics on SMADs 1, 2, and TAK1. Interestingly, among the numerous hits from the interactome screening, we noted αTAT1 as a potential binding partner for TAK1 with new functional implications (Supplementary Fig. 1). Subsequent biochemical studies confirmed their novel interaction as αTAT1 was pulled down upon TAK1 immunoprecipitation (Fig. 1a). A kinase-dead version of TAK1 (K63W point mutant) also bound efficiently with αTAT1, suggesting that the TAK1 catalytic activity is dispensable at least for their interaction (Fig. 1a), whereas parallel experiments showed that none of the SMAD proteins associated with this MT enzyme. To better understand the molecular basis of this interaction, pharmacologic inhibitors SB43152 and 5Z-7-Oxozeaenol were used to inhibit the kinases of TβRI and TAK1, respectively. Here, the TAK1/αTAT1 interaction was greatly enhanced upon inhibiting the activity of TAK1 but not TβRI kinase, indicating that αTAT1 may be a catalytic substrate for TAK1 (Fig. 1b). Notably, this interaction was also evident at the endogenous level where the two proteins readily co-localized and co-immunoprecipitated, but was further enhanced upon TGF-β stimulation (Fig. 1c, d). Lastly, to test whether their interaction is direct, binding studies were performed using recombinant purified proteins from the baculovirus/insect cell system (Fig. 1e and Supplementary Fig. 4). Here, purified biotin-labeled TAK1 was immobilized on streptavidin matrix and then co-incubated with purified αTAT1. Consistent with the co-immunoprecipitation data, αTAT1 was efficiently pulled down by biotin-labeled TAK1 to confirm their direct interaction (Fig. 1e).

Whether TGF-β controls the dynamics or PTMs of MTs in a rapid, non-transcriptional manner has never been reported. However, given our data demonstrating a direct interaction between TAK1 and αTAT1, the primary MT-acetylating enzyme in vivo, we explored the potential for TGF-β-induced TAK1 in directly modifying MT acetylation across cell types. Here, TGF-β caused a rapid increase in MT acetylation as observed by immunofluorescence, while the overall MT architecture remained constant (Fig. 1f, graphs). Accordingly, biochemical analysis showed rapid kinetics where TGF-β treatment markedly increased α-tubulin acetylation within 15 min and persisted up to 2 h, while total tubulin levels were unchanged (Fig. 1g). Next, as TGF-β signaling is mediated by the canonical TβRI/Smad2/3 and TAK1 pathways, cells were briefly treated with SB43152 or 5Z-7-Oxozeaenol and found that TAK1 inhibition reduced, whereas TβRI inhibition moderately enhanced MT acetylation, suggesting that TGF-β likely causes MT acetylation through TAK1 signaling (Fig. 1h, graphs).

**Fig. 2:**
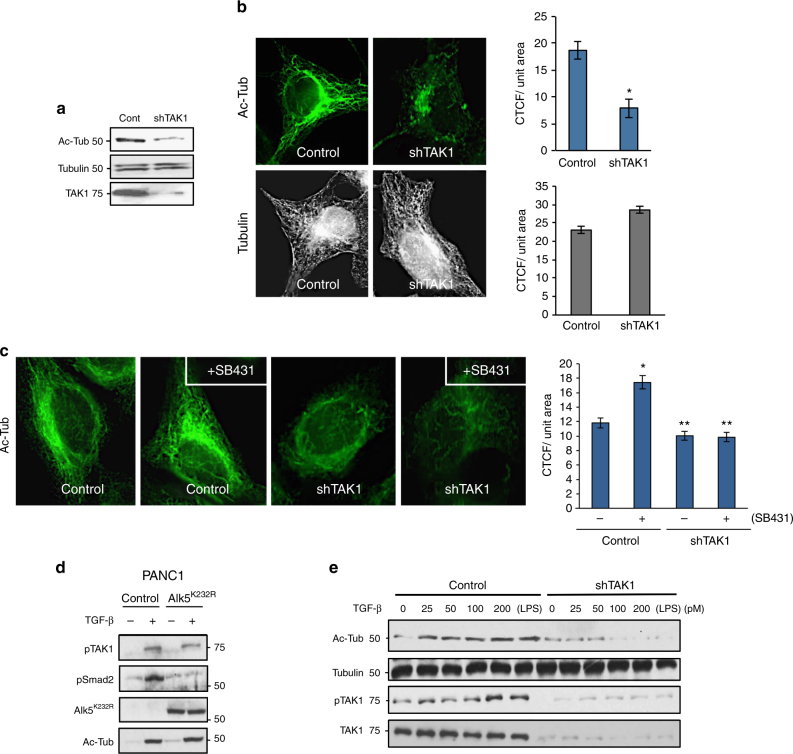
**TAK1 depletion abrogates TGF-β-induced MT acetylation. a** Western blot analysis of acetyl-tubulin, total tubulin, and total TAK1 in control HeLa and sh-TAK1 stable cells. **b** Control HeLa and sh-TAK1 stable cells imaged for acetyl tubulin and total tubulin immunofluorescence staining. The images were quantified for CTCF using Image J. At least 30 cells per condition were quantified, the images are representative of four independent experiments, *n* = 4. Error bars represent SEM and type 2 *t*-test show relative to control: *p < 0.001. **c** Control HeLa and sh-TAK1 stable cells were treated with SB431542 (30 μM) for 30 min before fixing and then imaged for acetyl tubulin immunofluorescence staining. The images were quantified for CTCF using Image J. At least 30 cells per condition were quantified. The images are representative of three independent experiments, *n* = 3, error bars represent SEM and type 2 *t*-test show relative to control: *p < 0.05, **p < 0.01. **d** Panc1 cells were transiently transfected with PCDNA control vector or myc-ALK5-K232R construct followed by treatment with TGF-β1 (200 pM; 1 h) before western blot analysis of phospho-TAK1, phospho-Smad2, ALK5-K232R, and acetyl tubulin. **e** Control HeLa and sh-TAK1 stable cells were treated with increasing concentrations of TGF-β1 (0, 25, 50, 100, and 200 pM) and 10 ng/ml LPS before western blot analysis of Acetyl-tubulin, total tubulin, phospho-TAK1, and total TAK1

To test whether TAK1 enhances acetylation directly or indirectly through its downstream effectors such as p38 and JNK, cells were preincubated with inhibitors of TAK1, p38 and JNK before TGF-β stimulation. Here, an almost twofold increase in acetylation was observed upon TGF-β treatment in control, while TAK1 inhibition fully abrogated the effects of TGF-β (Fig. 1i, graph). However, blocking p38 or JNK modestly increased basal MT acetylation independent of ligand treatment (Fig. 1i, graph), again supporting the likely direct role of TAK1 in MT acetylation. To further test this hypothesis, TAK1 stable knockdown cell lines (shTAK1) were generated to observe more than 70% reduction in basal MT acetylation over control cells as assessed by biochemical and immunofluorescence analyses (Fig. 2a, b, graph). Again, total tubulin levels and the overall MT morphology remained constant upon TAK1 depletion, indicating that TAK1 selectively targets MT acetylation (Fig. 2b, graphs).

Although the early events of TGF-β-induced TAK1 activation requires TAK1 to associate with TβRI, the TβRI kinase activity is surprisingly dispensable unlike the canonical Smad pathways that strictly require the receptor serine/threonine kinase to phosphorylate Smad2/3^19^. Therefore, we hypothesized that the enhanced MT acetylation previously observed upon SB43152 treatment (Fig. 1h) was largely due to greater access of TAK1 to TGF-β-bound TβRI than Smad2/3. Indeed, whereas MT acetylation was elevated upon TβRI kinase inhibition as previously observed in control cells (Fig. 1h), shTAK1 cells displayed strikingly reduced acetylation at basal state, but more importantly, failed to respond upon SB43152 treatment (Fig. 2c, graph). Expression of TβRI kinase-dead (ALK5^K232R^) also yielded similar results where TAK1 still induced MT acetylation in response to TGF-β while failing to activate Smad2 (Fig. 2d). Further substantiating this finding, a TGF-β dose–response study revealed a steady, concentration-dependent increase in both tubulin acetylation and TAK1 phosphorylation in control but not in shTAK1 cells (Fig. 2e). Similarly, we observed that LPS, a known TAK1 inducer, enhanced acetylation in a TAK1-dependent manner (Fig. 2e; lane 6). Taken together, these data strongly supported TAK1 as the principal mediator of TGF-β-induced MT acetylation.

**Fig. 3:**
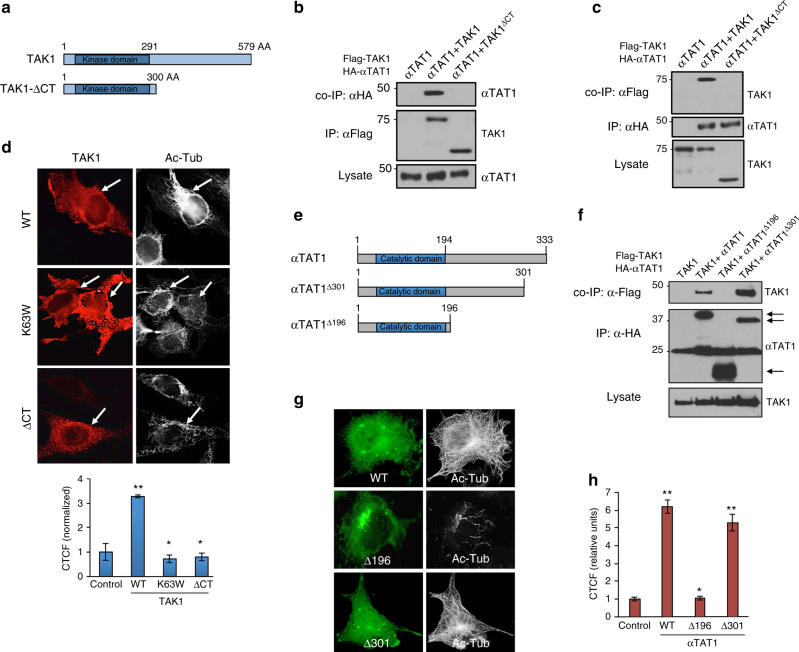
**Structural determinants of TAK1 required for αTAT1 binding. a** Schematic representation of TAK1 WT and C-terminal truncated mutant TAK1-ΔCT. **b** COS-7 cells expressing HA-αTAT1 WT, Flag-TAK1, or Flag- TAK1-ΔCT were immunoprecipitated with Flag antibody. **c** COS-7 cells expressing HA-αTAT1 WT, Flag-TAK1, or Flag-TAK1-ΔCT were immunoprecipitated with αHA antibody. **d** COS-7 cells expressing Flag-TAK1 WT, Flag-TAK1 KD (K63W), and Flag-TAK1-ΔCT were fixed and imaged for Flag (left panel) and Acetyl-tubulin (right panel) immunofluorescence co-staining. CTCF was quantified using Image J, at least 30 cells were quantified per condition. The images are representative of three independent experiments, *n* = 3. Error bars represent SEM and type 2 *t*-test analysis show relative to control: *not significant; **p < 0.001. **e** Schematic representation of αTAT1-WT and truncation mutants αTAT1-Δ301, αTAT1-Δ196. **f** COS-7 cells expressing Flag-TAK1 WT, HA-αTAT1 WT, HA-αTAT1-Δ196, or HA-αTAT1-Δ301 were immunoprecipitated with α-HA antibody. **g**, **h** COS-7 cells expressing HA-αTAT1 WT, HA-αTAT1-Δ196, or HA-αTAT1-Δ301 were fixed and imaged for α-HA (left panel) and Acetyl-tubulin (right panel) immunofluorescence co-staining. CTCF was quantified using Image J, at least 30 cells were quantified per condition. The images are representative of three independent experiments, *n* = 3. Error bars represent SEM and type 2 *t*-test analysis show relative to control: *not significant; **p < 0.001

Next, we sought to identify the critical structural determinants of the TAK1/αTAT1 complex by first testing a TAK1 C-terminal truncation mutant that retains the kinase domain (TAK1^ΔCT^) (Fig. 3a). Here, the immunoprecipitation of the full-length TAK1 strongly associated with αTAT1, whereas TAK1^ΔCT^ failed to interact (Fig. 3b). The reciprocal experiment in which αTAT1 was immunoprecipitated also yielded a consistent pattern, thus indicating that the TAK1 C-terminal domain is essential for their interaction (Fig. 3c). Notably, immunofluorescence data showed that TAK1-WT expressing cells conferred at least three-fold increase in MT acetylation compared with both the kinase-dead mutant TAK1^K63W^- and TAK1^ΔCT^-expressing cells, which remained at basal state similar to the surrounding non-transfected cells (Fig. 3d, arrows and graph).

To define the αTAT1 structural elements, we generated C-terminal deletions that truncate either the last 33 amino acids (αTAT1^Δ301^) or the last 137 residues that still retains the acetyltransferase catalytic domain (αTAT1^Δ196^) (Fig. 3e). Here, both the full-length and αTAT1^Δ301^ interacted efficiently with TAK1, whereas αTAT1^Δ196^ did not, indicating that the αTAT1 C-terminal region is not only critical for the interaction with TAK1 (Fig. 3f) but also required for robust MT acetylation such as the wild type (WT) (Fig. 3g, h).

### TAK1 phosphorylates αTAT1 to enhance its catalytic activity

**Fig. 4:**
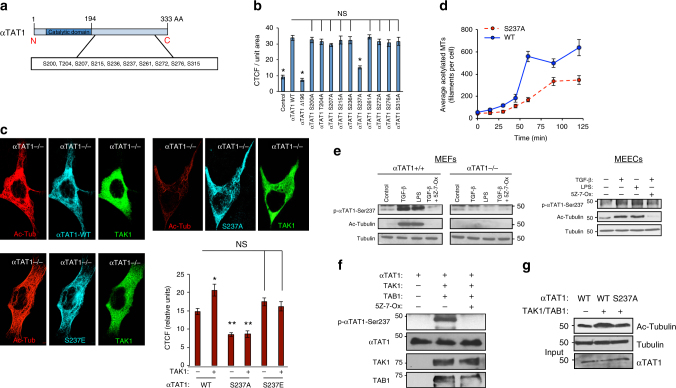
**αTAT1 catalytic activation requires direct phosphorylation at Ser237 by TAK1. a** Schematic representation of αTAT1 serine/threonine to alanine single point mutants. **b** COS-7 cells expressing αTAT1 WT or the serine/threonine to alanine single point mutants were fixed and imaged for αHA and Acetyl-tubulin. CTCF was quantified using Image J, at least 25 cells were quantified per condition. The images are representative of three independent experiments, *n* = 3. Error bars represent SEM and type 2 *t*-test analysis show relative to αTAT1 WT: *p < 0.001. **c** αTAT1^–/–^ MEFs expressing TAK1 WT or HA-αTAT1 WT, HA-αTAT1-S237A, and HA-αTAT1-S237E were fixed and imaged for Acetyl-tubulin (red), αHA (pseudo color cyan), and TAK1 (green) immunofluorescence co-staining. CTCF per unit area was quantified using Image J, at least 30 cells were quantified per condition. Error bars represent SEM and type 2 *t*-test analysis show relative to αTAT1 WT: *p < 0.001; **p < 0.0001. **d** COS-7cells expressing HA-αTAT1 WT or HA-αTAT1-S237A were subjected to nocodazole (100 nM) for 1 h, washed thoroughly with PBS, and then immediately fixed after 0, 15, 30, 45, 60, 90, and 120 min. The fixed cells were then imaged for αHA and Acetyl tubulin immunofluorescence co-staining. The images were quantified by skeletonizing the acetyl tubulin staining using Image J and skeletons analyzed for length and branching of acetyl tubulin filaments. **e** αTAT1^+/+^ MEFs, αTAT1^–/–^ MEFs, and mouse embryonic endothelial cells were treated with TGF-β (200 pM), LPS (10 ng/ml) for 30 min, or pre-treated with 5Z-7-oxozeaenol (20 μM) for 15 min followed by TGF-β stimulation. **f** In vitro kinase assay performed using purified αTAT1, TAK1, and TAB1 were incubated with and without treatment with 5Z-7-oxozeaenol (20 μM). **g** In vitro acetylation assay performed using purified tubulin, αTAT1, αTAT1-S237A, TAK1, and TAB1

Based on these findings, we tested whether TAK1 mediates MT acetylation either by promoting αTAT1 trafficking/recruitment to the MTs or through direct phosphorylation-induced enzyme activation. To address the former possibility, we assessed the endogenous αTAT1 co-localization with MTs in control and shTAK1 HeLa cells (Supplementary Fig. 2). Here, αTAT1 co-localized within MTs efficiently independent of TAK1 depletion or TGF-β stimulation (Supplementary Fig. 2, graph), thereby suggesting that TAK1 does not help recruit αTAT1 to MTs. This finding prompted us to focus on the C-terminal region of αTAT1 as a potential phosphorylation target, as it was deemed critical for its interaction with TAK1 and MT acetylation (Fig. 4a). Initial attempts to identify novel TAK1-induced phosphorylation sites through mass spectrometry proved unsuccessful due to a combination of insufficient sequence coverage and inability to differentiate adjoining serine phosphorylation sites (e.g., Ser236 and Ser237). Further hindering our efforts was the fact that TAK1 phosphosubstrates lack a conserved phosphorylation motif; hence, we systematically mutated every serine and threonine residue within this C-terminal region (residues 200–315) to alanine via site-directed mutagenesis and functionally screened for their MT-acetylating capacity (Fig. 4b). Relative to control at basal state, the WT αTAT1-expressing cells yielded greater than threefold enhancement, whereas αTAT1^Δ196^-expressing cells had no effect similar to control as expected (Fig. 4b). However, among the ten additional αTAT1 point mutants tested, all yielded remarkably similar levels of MT acetylation relative to WT with the exception of αTAT1^S237A^ (Fig. 4b).

Hence, to further interrogate the functional role of this phosphorylation site, phosphomimetic constructs (Asp D and Glu E) were generated and each expressed in αTAT1-knockout (αTAT1^–/–^) mouse embryonic fibroblasts (MEFs). Whereas αTAT1^–/–^ MEFs were devoid of MT acetylation as expected (Supplementary Fig. 3A), the ectopic expression of the WT and those of Ser237D and Ser237E mutants produced robust MT acetylation, whereas Ser237A displayed significantly impaired catalytic activity in both αTAT1^–/–^ MEFs and COS-7 cells (Fig. 4c, graph). Notably, co-expressing TAK1 led to greater acetylation in WT but not Ser237E or the Ser237A mutant, suggesting that TAK1 acts specifically through this phosphorylation site (Fig. 4c, graph). Similar effects were observed in other cell types including COS-7 cells wherein overexpression of WT and S237D and E, but not S237A, dramatically enhanced MT acetylation over basal non-transfected cells (Supplementary Fig. 3B, graph). Furthermore, we observed that TGF-β stimulation enhanced, whereas 5Z-7-Oxozeaenol attenuated MT acetylation in WT αTAT1-expressing cells, while those expressing αTAT1^S237A^ failed to respond to either TGF-β or 5Z-7-Oxozeaenol (Supplementary Fig. 3C).

However, to exclude the possibility of adaptive mechanisms contributing to MT acetylation, we measured the kinetics of the WT or αTAT1^S237A^ upon brief nocodazole treatment to synchronize and abolish MT acetylation (Fig. 4d). Here, subsequent washout allowed for the visualization of steadily growing MTs and their progressive acetylation at various time points. During the 2 h time course, maximum MT acetylation levels were achieved and then plateued within 1 h in the WT, whereas αTAT1^S237A^ lagged far behind at the 1 h mark and beyond, reaching roughly 50% capacity of the WT (Fig. 4d). Hence, these findings strongly supported the TAK1 phosphorylation of αTAT1 on site Ser237 as a key mechanism of MT acetylation.

In order to prove αTAT1-Ser237 as a direct phosphorylation site targeted by TAK1, we raised a custom phospho-specific antibody. However, upon careful testing the antibody was determined to be incompatible with western blotting yet suitable for enzyme-linked immunosorbent assays and immunoprecipitation. Thus, we first assessed whether endogenous αTAT1 becomes phosphorylated at Ser237 upon stimulation with either TGF-β or LPS followed by immunoprecipitation with the phosphoantibody, and then immunoblotted with pan-αTAT1 antibody. In both MEFs and a mouse endothelial cell line, both TGF-β and LPS induced MT acetylation as expected and, more importantly, was accompanied by the detection of phosphorylated αTAT1-Ser237 (Fig. 4e). To further demonstrate that these were TAK1-directed effects, cells were pre-incubated with TAK1 inhibitor before TGF-β stimulation and found that αTAT1-Ser237 phosphorylation was abrogated along with MT acetylation (Fig. 4e).

To provide definitive evidence of direct TAK1-induced effects on αTAT1-Ser237 phosphorylation, we turned to recombinant purified proteins in a cell-free system. In most cellular contexts, TAK1 associates with TAK1-binding protein (TAB1) upon TGF-β stimulation to activate the serine/threonine kinase. Although in our co-precipitation studies TAB1 proved to be dispensable for the TAK1/αTAT1 interaction, we generated purified TAK1, TAB1, and αTAT1 proteins, and performed an in vitro kinase assay in the presence or absence of 5Z-7-Ox preincubation. Here we observed a robust αTAT1-Ser237 phosphorylation with the addition of the TAK1/TAB1 complex, whereas the same complex failed to phosphorylate αTAT1-Ser237 in the presence of 5Z-7-Ox (Fig. 4f). Likewise, the recombinant TAK1/TAB1 complex was capable of further acetylation of purified α-tubulin in the presence of WT but not αTAT1-Ser237 (Fig. 4g). Collectively, these results strongly support αTAT1 as a novel TAK1 substrate requiring Ser237 phosphorylation for enhancement of αTAT1 catalytic activity.

### αTAT1 regulates cell growth and maintenance by AKT inactivation

**Fig. 5:**
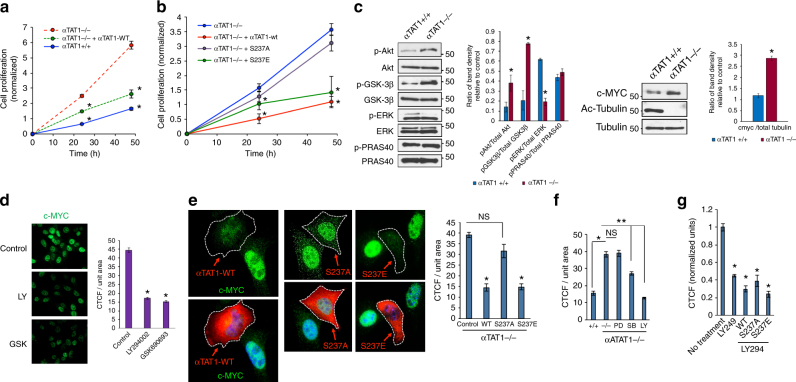
**αTAT-Ser237 phosphorylation regulates cell proliferation. a** Crystal Violet growth assay of αTAT1^+/+^ MEFs, αTAT1^–/–^ MEFs, and αTAT1^–/–^ MEFs rescued with ectopic expression of αTAT1 WT over the period of 24 and 48 h. Presented data are average of four independent experiments. Data were analyzed by two-way ANOVA with Bonferroni post-hoc test: *p < 0.05. **b** Crystal Violet growth assay of αTAT1^–/–^ MEFs, αTAT1^–/–^ MEFs rescued with ectopic αTAT1 WT, S237A, or S237E. Presented data is average of three independent experiments. Data were analyzed by two-way ANOVA with Bonferroni post-hoc test: *p < 0.05. **c** Western blot analyses demonstrating levels of pERK, total ERK, pAKT S473, total AKT, c-MYC, pGSK3β, total GSK3β, pPRAS40, total PRAS40, acetyl tubulin, and total tubulin in αTAT1^+/+^ and αTAT1^–/–^ MEFs. **d** αTAT1^–/–^ cells were treated with PI3K inhibitor LY294002 (20 μM) or pan AKT inhibitor GSK690693 (10 µM) for 1 h, fixed and imaged for c-MYC immunofluorescence staining. CTCF per unit area was quantified using Image J, at least 60 cells were quantified per condition. Error bars represent SEM and type 2 *t*-test analysis show relative to αTAT1^–/–^ control: *p < 0.05. **e** αTAT1^–/–^ cells expressing HA-αTAT1 WT, HA-αTAT1 S237A, or HA-αTAT1 S237E were fixed and imaged for αHA (red) and c-MYC (green) immunofluorescence co-staining. CTCF per unit area was quantified using Image J, at least 20 cells were quantified per condition. Error bars represent SEM and type 2 *t*-test analysis show relative to control: *p < 0.00001, NS (*p* = 0.3). **f** αTAT1^–/–^ cells were treated with ERK1 inhibitor PD98059 (20 μM), p38 inhibitor SB203580 (15 μM), and PI3K inhibitor LY294002 (20 μM) for 30 min, fixed, and imaged for c-MYC immunofluorescence staining. CTCF per unit area was quantified using Image J, at least 60 cells were quantified per condition. Error bars represent SEM and type 2 *t*-test analysis show relative to αTAT1^+/+^ control: *p < 0.05 and relative to αTAT1^–/–^ control: **p < 0.05. **g** αTAT1^–/–^ cells were rescued with ectopic expression of HA-αTAT1 Wt, HA-αTAT1 S237A or HA-αTAT1 S237E and were treated with LY294002 (20 μM) for 30 min before fixing. The conditions were imaged for αHA (red) and c-MYC (green) immunofluorescence co-staining. CTCF per unit area was quantified using Image J, at least 30 cells were quantified per condition, normalized with respect to control condition. Error bars represent SEM and type 2 *t*-test analysis show relative to normalized control: *p < 0.0001

Although αTAT1 can suppress cell growth through contact inhibition^26^, still a crucial question is whether it acts through MT stabilization, thereby impairing such process as mitotic spindle assembly, or through regulation of intracellular transport of key intracellular signaling molecules. To test this and understand how TGF-β contributes to this process, we focused on the role of TAK1 phosphorylation of αTAT1 and MT acetylation in cell growth. As previously reported, αTAT1^–/–^ MEFs proliferated more rapidly over the course of 48 h than control cells, a trend that was reversed upon rescue expression of the αTAT1 WT (Fig. 5a; 24–48 h). To test whether αTAT1 inhibits cell growth mainly through MT acetylation, and further dissect the role of αTAT1-Ser237 phosphorylation, αTAT1^–/–^ MEFs were transfected with the WT, Ser237A, and Ser237E (Fig. 5b). Here, both the αTAT1^–/–^ MEFs and Ser237A-expressing cells had greater rate of proliferation than the WT or the phosphomimetic, suggesting that Ser237 phosphorylation critically regulates cell growth (Fig. 5b). This growth pattern was similarly evidenced in epithelial cell types including HeLa, COS-7, and PANC1 where WT and S237E suppressed cell proliferation over control cells, whereas αTAT1^S237A^ expression had minimal impact even in the presence of TAK1 coexpression (Supplementary Fig. 5A-C).

As we observed minimal changes in the overall MT architecture, we reasoned that acetylation might have a more prominent role in the intracellular transport of mitogenic signaling molecules. Various growth-related signaling pathways therefore were screened in control and αTAT1 knockout cells including ERK, p38, JNK, and AKT along with many other signaling effectors of cell growth and survival (Fig. 5c, graphs). Surprisingly, most growth-promoting pathways remained relatively constant or were only modestly affected, with the exception of p38 and AKT (Fig. 5c and Supplementary Fig. 5D). Although follow-up αTAT1 rescue in αTAT1^–/–^ MEFs ruled out p38 as being independent of cell growth or MT acetylation, AKT activity was distinctly negatively regulated by αTAT1 expression (Fig. 5c). More notably, the enhanced AKT activation in αTAT1-null cells led to the prominent phosphorylation of a key downstream substrate, GSK3β, but not PRAS40, another substrate involved in proliferation but is largely insulin-responsive, suggesting that αTAT1 regulation of AKT signaling is highly selective during cell growth suppression (Fig. 5c). Consistent with this notion, we found that GSK3β-inactivating phosphorylation promoted c-MYC levels, a major driver of cell cycle progression (Fig. 5c). Indeed, previous work has shown that AKT phosphorylation of GSK3β inactivates its kinase activity towards c-MYC at Thr58, a site that promotes c-MYC proteasomal degradation upon phosphorylation. To determine whether c-MYC elevation is largely dependent on this pathway, αTAT1^–/–^ MEFs were treated with an inhibitor of PI3-Kinase (LY294002) or Akt (GSK690693). As observed by immunofluorescence, both PI3-Kinase and Akt inhibition suppressed c-MYC expression relative to control (Fig. 5d). The role of αTAT1 in regulating the c-MYC level was also evident, as reconstituting the expression of αTAT1 WT, S237A, or S237E in αTAT1^–/–^ cells showed c-MYC levels being strongly suppressed in the WT and S237E but not S237A-expressing cells (Fig. 5e and Supplementary Fig. 5E, and graphs). In parallel experiments, we quantified these results upon blocking ERK, p38 and PI3K/AKT activity and found reduced c-MYC levels comparable to WT upon blocking PI3K activation, suggesting that αTAT1 suppresses AKT activation by at least partially inhibiting its recruitment to the membrane (Fig. 5f), a finding that was further substantiated by the fact that both the αTAT1^–/–^ and S237A expressing cells failed to augment c-MYC expression when the PI3K/AKT pathway was blocked (Fig. 5g).

**Fig. 6:**
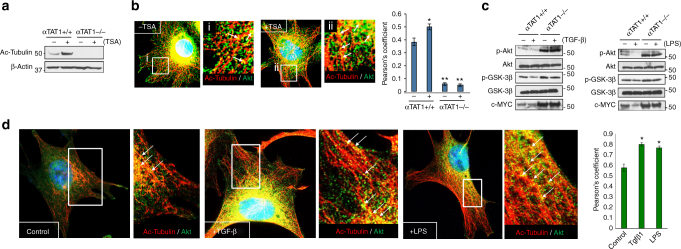
**MT acetylation inhibits AKT activation. a** Western blot analyses demonstrating acetyl tubulin level post Trichostatin A (TSA; 500 nM) treatment for 30 min in αTAT1^+/+^ and αTAT1^–/–^ MEFs. **b** αTAT1^+/+^ and αTAT1^–/–^ MEFs were treated with 500 nM of TSA for 30 min before fixing and immunofluorescence staining for acetyl tubulin (red) and total Akt (green). Representative images are obtained from αTAT1^+/+^ MEFs with and without TSA treatment. Acetyl tubulin levels were undetectable in αTAT1^–/–^ cells. Pearson’s correlation coefficient was calculated to determine the proximity between acetyl tubulin and total Akt. Image J plugin called Just another co-localization plugin (JACoP) was used to quantify the images. Thirty cells per condition and three ROIs per cell were quantified. Error bars represent SEM and type 2 *t*-test analysis show relative to αTAT1^+/+^ control: *p < 0.05, **p < 0.0001. **c** αTAT1^+/+^ and αTAT1^–/–^ MEFs were treated with TGF-β1 (200 pM; 30 min) or LPS (10 ng/ml; 30 min) before western blot analyses of the lysates for levels of pAKT S473, total AKT, pGSK3β, total GSK3β, and c-MYC. **d** Immunofluorescence images demonstrating localization of acetyl tubulin (red) and total Akt (green) in αTAT1^+/+^ MEFs post treatment with TGF-β1 (200 pM; 30 min) or LPS (10 ng/ml; 30 min). Pearson’s correlation coefficient was calculated using JACoP, 15 cells per condition, and 3 ROIs per cell were quantified. Error bars represent SEM and type 2 *t*-test analysis show relative to αTAT1^+/+^ control: *p < 0.05

Many signaling molecules including AKT have been shown to associate with MTs as a mode of intracellular transport. We hypothesized that acetylation sequesters MT-bound AKT, thereby preventing access to the plasma membrane for activation. To test this, control and αTAT1^–/–^ MEFs were first treated with Trichostatin A (TSA), a broad pharmacologic inhibitor of deacetylases, and found a substantial increase in WT MEFs, whereas αTAT1^–/–^ cells were devoid of tubulin acetylation as expected (Fig. 6a). Similarly, immunofluorescence studies revealed prominent co-localization of acetylated MTs with endogenous AKT at basal state, which was further enhanced upon TSA treatment (Fig. 6b arrows and Supplementary Fig. 6A). Moreover, MT acetylation inversely correlated with AKT activation in which αTAT1^–/–^ cells, devoid of MT acetylation, produced greater AKT activation irrespective of TSA, whereas WT cells showed diminished activity upon treatment (Supplementary Fig. 6B). More importantly, AKT activation along with GSK3β and c-MYC levels strongly correlated with TAK1 activity, as both TGF-β and LPS attenuated these pathways in WT but not in αTAT1^–/–^ MEFs (Fig. 6c), findings consistent with AKT retention along acetylated MTs to prevent intracellular transport to the plasma membrane for activation. In fact, as assessed by both co-immunoprecipitation and immunofluorescence, this selective association between AKT and acetylated MTs was greatly enhanced in WT but not αTAT1^–/–^ MEFs upon treatment with TAK1 inducers (i.e., TGF-β and LPS) and even paclitaxel, an inhibitor of MT depolymerization that typically causes MT acetylation (Fig. 6d and Supplementary Fig. 6C).

**Fig. 7:**
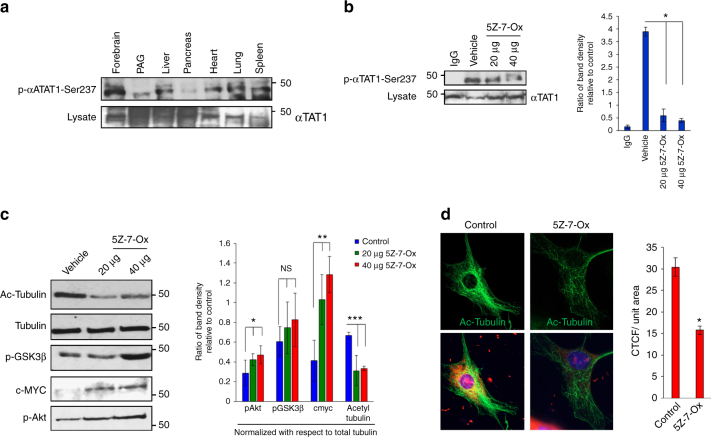
**TAK1-dependent αTAT1 activity in the brain. a** Western blot analyses demonstrating phosphorylation levels on serine-237 residue of αTAT1 in lysates extracted from mouse tissues such as forebrain, periaqueductal gray (PAG), liver, pancreas, heart, lung, and spleen. **b** Representative western blottings demonstrating phospho-αTAT1-Ser237 and total αTAT1 levels in lysates extracted from mice (*n* = 4) with and without treatment with TAK1 inhibitor 5Z-7-oxozeaenol (20 µg or 40 µg per animal; 1 h). Band density of four independent blots was calculated using ImageJ. Error bars represent SEM and type 2 *t*-test analysis show relative to control: *p < 0.05. **c** Representative western blottings demonstrating acetyl tubulin, total tubulin, pGSK3β, cMYC, and pAKT-S473 levels in lysates extracted from mice (*n* = 4) with and without treatment with TAK1 inhibitor 5Z-7-oxozeaenol (20 µg or 40 µg per animal; 1 h). Band density of four independent blots was calculated using ImageJ. Error bars represent SEM and type 2 *t*-test analysis show relative to control: *p < 0.05 (pAKT), **p < 0.05 (cMYC), ***p < 0.05 (acetyl tubulin), NS is not statistically significant (pGSK3β). **d** Immunofluorescence images demonstrating acetyl tubulin (green) levels in neurons (red, Nissl stain) extracted from the dorsal root ganglia (DRG) of mice. The neurons were subjected to TAK1 inhibitor 5Z-7-oxozeaenol (20 µM; 1 h), 24 h post dissection. CTCF per unit area was quantified using Image J, at least 40 cells were quantified per condition. Error bars represent SEM and type 2 *t*-test analysis show relative to control: *p < 0.05

Finally, in order to gain novel functional insights in vivo, we screened for relative αTAT1 catalytic activity in mouse using our novel phospho-Ser237 antibody and found that large portions of the brain contained some of the highest levels among the various tissue types (Fig. 7a). Here, TAK1 was inhibited through intracerebroventricular injection of 5Z-7-Ox and subsequently analyzed for p-αTAT1-Ser237 levels. There was a dose-dependent decrease in phosphorylation (Fig. 7b) as predicted, although more strikingly, MT acetylation was dramatically reduced while p-AKT, p-GSK3β, and c-MYC levels all increased (Fig. 7c, graph). Similarly, freshly isolated dorsal root ganglion (DRG) cells briefly exposed to 5Z-7-Ox also displayed significantly diminished MT acetylation (Fig. 7d). As illustrated in Supplementary Fig. 7, these combined in vitro and in vivo results provide compelling evidence for the role of TAK1 in αTAT1 regulation of GSK3β and c-MYC signaling through MT-based AKT sequestration and inactivation.

## Discussion

As canonical mediators of TGF-β signaling, receptor-activated SMADs produce pleiotropic effects by shuttling to the nucleus to regulate the expression of hundreds of target genes in a highly cell-type and context-specific manner^13,27^. This requires SMAD proteins to associate with various kinesin and dynein motor proteins to move along the microtubular network^28,29^. In the present study, we report on what is believed to be the first non-transcriptional regulation of MT functions by TGF-β signaling mediated by rapid TAK1 activation of αTAT1. These findings have broad and significant pathophysiologic implications as aberrant TGF-β signaling through TAK1 underlies numerous conditions including cancer, inflammatory and neurologic disorders. Although beyond the scope of the present study, future exploration of how TGF-β-related changes in αTAT1 function and MT acetylation contribute to these cellular and disease states will be vitally important as new TGF-β-targeted therapies continue to emerge in clinical settings.

Very recently, TGF-β has been shown to modulate MT acetylation during epithelial-mesenchymal transition^30,31^. Although the mechanisms are still being investigated, they appear to be based on context-specific transcriptional events, as acetylation reportedly occurs in the course of 48 h in epithelial cells. Our central findings represent a broader scale as we observed TAK1-induced MT acetylation in response to TGF-β1 that persists for at least 2 h across multiple cell types including mouse fibroblasts, human epithelial, and endothelial cells. It is also noteworthy that similar effects were observed upon stimulation with LPS, another potent inducer of TAK1 tied to inflammatory process.

The precise role of tubulin acetylation in MT stability has long remained controversial, as various genetic, cellular, and in vitro findings have ranged from only minor effects to being required for the overall MT integrity^4^. However, most recent data provide compelling new evidence in support of structural stability, as acetylation appears to protect long-lived MTs against mechanical ageing and resistance to mechanical breakage^32,33^. Although our work did not specifically examine these aspects, we found no discernable signs of breakage or depolymerization of existing MTs upon depletion or inhibition of TAK1 activity, nor in response to the overexpression of the various αTAT1 constructs. This finding surprisingly contrasts with at least one report in which αTAT1 overexpression was shown to destabilize MTs through its interaction rather than its acetyltransferase activity^34^. Instead, our data support the primary role of TAK1 in regulating αTAT1 enzymatic activity rather than its recruitment to or physical association with MTs.

Over the years, numerous cellular stresses have been shown to induce MT acetylation including UV irradiation, osmotic, immune, and viral responses. Hence, it is not surprising that αTAT1 is also modulated by various stress stimuli, although in most cases the underlying mechanisms remain poorly defined. In fact, with the exception of one previous study examining the role of ROS-mediated AMPK activation, little is known about how αTAT1 is regulated. In this study, ROS-mediated AMPK activation reportedly enhanced αTAT1 activity through phosphorylation, though whether αTAT1 is a direct AMPK phosphosubstrate remains uncertain^12^.

Interestingly, given that TAK1 can activate AMPK in many contexts, it remains to be determined whether the ROS-AMPK pathway controlling αTAT1 requires TAK1 activity. Nevertheless, it seems likely to be that TAK1 regulation of αTAT1 can occur independently of AMPK, as our work demonstrates that the TAK1/αTAT1 interaction is direct and that Ser237 phosphorylation occurs even in an in vitro setting using purified proteins. Still, considering the fact that TAK1 is activated by such diverse stress stimuli across cell types, it will be important to fully characterize the relationship between TAK1 and AMPK activity in regulating αTAT1 and MT acetylation.

MTs have a key role in intracellular transport of protein cargo through kinesin-1 and kinesin-like motor proteins, which preferentially bind to stable MTs marked by acetylation and detyrosination^5^. In our studies, genetic and pharmacologic manipulation of tubulin acetylation revealed that MT acetylation has a strong sequestering effect rather than dynamic transport of AKT. Based on our data, it is likely to be that AKT sequestration is at least partially selective, as mitogenic and other cell growth-related pathways seem largely unaffected by MT acetylation.

A notable observation was that, contrary to our expectation, there were no αTAT1 or MT acetylation-dependent changes in TGF-β-responsive Smad 2/3 activation even though it is a potent inhibitor of cell growth that also requires MT-based transport. Taken together, our findings support a working model in which TGF-β-responsive TAK1 phosphorylates and activates αTAT1 to enhance MT acetylation, which in turn, preferentially binds to and sequesters AKT to prevent its access to the plasma membrane where it normally becomes activated.

Although our in vivo findings demonstrate that TAK1 activation of αTAT1 and its downstream impact on AKT signaling goes beyond cultured cell lines, the pathophysiologic outcome of altering TAK1 activity particularly in the brain may prove much more complex and warrants further investigation. Indeed, given that αTAT1-null mice are viable yet exhibit cognitive dysfunctions, it is possible that only certain regions of the brain or neuronal subtypes that depend more highly on TAK1 activity are susceptible to neurodegenerative dysfunction.

In summary, the present work establishes a fundamental new role for TGF-β in regulating MT functional dynamics. Activation of TAK1 by TGF-β superfamily ligands as well as LPS can cause phosphorylation-induced αTAT1 activation and MT acetylation. Our findings reveal novel pathophysiologic implications between TGF-β signaling and aberrant MT acetylation in cancer and neurodegenerative disorders.

## Methods

### Antibodies

The following antibodies used in this study were purchased from Cell Signaling: acetyl-α-tubulin (1:1000; #5335), TAB1 (1:1000; #3226), pERK (1:2000; #9101), total ERK (1:2000; #4695), pAKT-S473 (1:1000; #4060), pP38 (1:2000; #4511), pJNK (1:2000; #9255), cMYC (1:2000; #13987), pGSK3β-Ser9 (1:2000; #5558), total GSK3β (1:2000; #12456), pTAK1 (1:1000; #9339), total TAK1 (1:1000; #4505), total AKT (1:1000; #4691), pPRAS40 (1:2000; #2997), total PRAS40 (1:2000; #2610), pSmad2 (1:1000; #8828), and Myc-Tag (1:4000; #2276). Total tubulin YOL1/34 (1:2000; MCA78G) (Abcam), αHA (1:3000; #11867431001), α-Flag (1:4000; F1804) (Sigma), αTAT1 phosphoserine 237 (1:250) (Thermo Scientific), and αTAT1 (1:500; sc-167354) (Santa Cruz) were also used.

### Animal studies

Male CD-1 (ICR) mice in age-matched controlled cohorts from 4 to 8 weeks of age were used for all experiments and were obtained from Charles River Laboratories (Wilmington, MA). Mice were kept in an animal facility at University of Arizona, no more than five mice per cage, under environmental temperature and humidity control with 12 h light/dark cycles and free access to food and water. All experiments performed were in accordance with Institutional Animal Care and Use Committee-approved protocols of the University of Arizona. For the dose–response of TAK1 inhibitor 5Z-7-oxozeaenol experiments, the mice were injected I.C.V. with 20 or 40 µg of 5Z-7-Ox or vehicle into left ventricle of the brain for 1 h before killing by rapid cervical dislocation. The brain was removed and rapidly frozen on liquid nitrogen before use. For DRG isolation, the spines from freshly sacrificed mice were dissected out and DRGs were extracted from the spinal cord and stored in the DRG+ media (Dulbecco’s modified Eagle’s medium (DMEM), fetal bovine serum (FBS), and Normocin) before incubation in collagenase/protease mixture for 1 h at 37 °C. Post-incubation DRGs were washed and reincubated in DRG+ media containing NGF (30 ng/µl). The DRGs were plated on poly-d-lysine-coated coverslips for immunofluorescence studies.

### Plasmid, constructs, and shRNAs

The GFP-αTAT construct was obtained from Addgene (Plasmid #27099; Dr. Maxence Nachury) and Flag-TAK1, Flag-TAK1-K63W, and TAB1 constructs were generous gifts from Dr. Jianhua Yang (Baylor College of Medicine). The Flag-Tak1Δ300 mutant was generated by PCR amplification using Flag-TAK1-WT as a template and forward and reverse primer complementary to the sequences near the defined regions. The HA-αTAT construct was generated by PCR amplification using GFP-αTAT as a template. HA-αTATΔ196 and Δ301 contstructs were generated by using HA-αTAT-WT as template with forward and reverse primers complementary to the sequences near the defined regions. HA-αTAT point mutations (S200A, T204A, S207A, T215A, S236A, S237A, T261A, S272A, S276A, S315A) were generated by site directed mutagenesis using HA-αTAT-WT as template and specific forward primer for each and reverse primer. TAK1 stable knockdown cells were generated in Hela cells was achieved by transfecting cells separately with two shRNA vector purchased from sigma (Mission shRNA # TRCN0000001556, (5′-CCGGCAGTGTGTCTTGTGATGGAATCTCGAGATTCCATCACAAGACACACTGTTTTT-3′) and

# TRCN0000001557, (5′-CCGGGACACACATGACCAATAACAACTCGAGGTTGTTATTGGTCATGTGTGTCTTTTT-3′)

Transfected cells were selected in media containing 3 mg/mL puromycin, and stable colonies with Smad2 depletion were confirmed by western blotting.

### Cell culture and transfection

COS-7, HeLa, and MEFs were cultured in DMEM (GIBCO) supplemented with 10% FBS (HyClone). Mouse embryonic endothelial cells were maintained in MCDB-131 (GIBCO) supplemented with 10% FBS, 2 mM l-glutamine, endothelial cell growth supplement (Sigma). αTAT 1^–/–^ MEFs were a generous gift from Dr. Maxence Nachury (Stanford). HeLa sh-TAK1 knockdown stables were generated first by small hairpin RNA vector transfection, selected in puromycin (1–2 mg/mL), then colonies were isolated and biochemically validated for TAK1 knockdown. Transfection was performed using Lipofectamine 2000 (Invitrogen).

### Immunoprecipitation

Cells were washed with phosphate-buffered saline (PBS), lysed on ice with lysis buffer for 20 min (20 mM HEPES pH 7.4, 150 mM NaCl, 5 mM NaF, 1 % NP-40) before centrifugation at 13,000 r.p.m. for 13 min. Supernatants were incubated with appropriate antibodies and agarose G or protein A agarose for 6 h at 4 C. Immunoprecipitates were then pelleted and washed three times before storing them in 2 × sample buffer followed by western blot analyses.

### Immunofluorescence studies

Cells grown on gelatin or poly-l-lysine (GIBCO) coated coverslips were fixed with 4% paraformaldehyde, permeabilized in 0.1 % Triton X-100 in PBS for 4 min, then blocked with 5% bovine serum albumin in PBS for 20 min. The primary antibody and fluorescently conjugated secondary antibodies were incubated at room temperature for 1.5 h. The cover slips were mounted using anti-fade that contained 4',6-diamidino-2-phenylindole. Confocal Imaging was performed on Nikon A1R laser microscope.

### Crystal violet growth assay

αTAT1^+/+^ or αTAT1^–/–^ was plated at a density of 10,000 cells per well in 12-well plates. HeLa scrambled control or HeLa shTAK1 were plated at a density of 15,000 cells per well in 12-well plates. Conditions involving ectopic expression of αTAT1 or TAK1 constructs were fixed (4% paraformaldehyde for 17 min) at different time points starting at 24 h post transfection. Following fixation, cells were washed with 1 × water and stained with 0.1% crystal violet for 30 min. Cells were washed with 1 × miili Q water repeatedly and air dried for 30 min. Cells were de-stained using crystal violet de-staining solution (10% acetic acid, 50% methanol, 40% H_2_O) for 15 min and the optical density was read at 590 nm in a microplate reader.

### Cloning, generation of recombinant baculoviruses, and transfection

Genes coding for full-length αTAT, TAK1, and TAB1 were cloned in a pFastBac™-Dual vector (Life Technologies). αTAT was cloned between the EcoR1 and Hind III restriction site from a DNA fragment produced by PCR using the WT gene as template, amplified with 5′- and 3′-hexahistidine-tagged primer (5′-GC GAA TTC GCC ATG GGT ATG CAC CAC CAC CAC CAC CAC GAG TTC CCG TTC GAT GTG-3′ and 5′-CCC AAG CTT TTA GTA TCG ACT CTC CTC-3′). TAK1 was cloned between EcoR1 and Kpn1 restriction site from a DNA fragment produced by PCR using the WT gene as template, amplified with 5′- and 3′-hexahistidine-tagged primer (5′-GC GAA TTC GCC ATG GGT ATG CAC CAC CAC CAC CAC CAC TCT ACA GCC TCT GCC GCC-3′ and 5′-CGG GGT ACC TCA TGA AGT GCC TTG TCG-3′). TAB1 was cloned between the EcoR1 and Hind III restriction site from a DNA fragment produced by PCR using the WT gene as template, amplified with 5′- and 3′-hexahistidine-tagged primer (5′-GC GAA TTC GCC ATG GGT ATG CAC CAC CAC CAC CAC CAC GCG GCG CAG AGG AGG AGC-3′ and 5′-CCC AAG CTT CTA CGG TGC TGT CAC CAC-3′). Once constructed, each plasmid was checked by sequencing and colony-PCR and used to transform MAX Efficiency® DH10Bac™ competent cells for production of recombinant bacmids by using Bac-to-Bac® Baculovirus Expression System. Transfection was performed in SF21 insect cell line by using Cellfectin II reagent (Invitrogen) according to protocols provided by manufacturer. Purified recombinant viruses were amplified with three rounds of infection in Sf21 cells grown at 27 °C using a multiplicity of infection (MOI) of 0.1. Viral supernatants were harvested 48–72 h post infection.

### Purification of recombinant proteins

Sf21 cells were infected with baculovirus at an MOI of 2. After 48 h post infection, cells were collected by centrifugation at 1000 × *g* for 3 min, gently washed with resuspension buffer (20 mM HEPES at pH 7.4, 0.5 M NaCl, 250 mM sucrose, a protease inhibitors [5 µg/ml aprotinin, 5 µg/ml leupeptin, 2 µM pepstatin A, 1 mM PMSF]). Cells were again plated and then re-suspended with sucrose free resuspension buffer (minus sucrose) and then lysed using a sonication (Vibro Sonics) at 50% amplitude with 5 s on and 5 s off for 10 min. Sonication and all subsequent steps were performed in 4 °C or on ice. After sonication, the cell lysate was centrifuged at 11,000 r.p.m. for 20 min and the supernatant was collected and supplemented with 0.05% Triton X-100. The cell-free supernatant was loaded onto HisPur cobalt resin column (Thermo Scientific) pre-equilibrated with resuspension buffer (10 ml). The column was washed first with 30 ml of buffer (20 mM HEPES (pH 7.4), 0.5 M NaCl, 12 mM imidazole, and 0.05% Triton X-100), then washed with additional 10 volumes of TritonX-100-free wash buffer. Recombinant Hexahistidine-tagged proteins were eluted with buffer containing 20 mM HEPES (pH 7.4), 0.5 M NaCl, 150 mM Immidazole (20 ml), and 0.5 ml fractions were collected. Protein fractions were resolved on 10% SDS-polyacrylamide gel electrophoresis (PAGE). The fractions containing purified protein were pooled and dialyzed extensively against 20 mM HEPES (pH 7.4) 100 mM NaCl at 4 °C^35^.

### Nocodazole-based kinetics

COS-7 cells expressing HA-αTAT1 WT or HA-αTAT1-S237A were subjected to nocodazole (100 nM) for 1 h, washed thoroughly with PBS, and then immediately fixed after 0, 15, 30, 45, 60, 90, and 120 min. The fixed cells were then imaged for αHA and Acetyl tubulin immunofluorescence co-staining. The images were quantified by skeletonizing the acetyl tubulin staining using Image J and skeletons analyzed for length and branching of acetyl tubulin filaments.

### In vitro kinase assay

Kinase assays were typically performed at 25 °C by preincubating a 0.5 µM concentration of protein in 1 mM MnCl2 for 5 min in buffer (20 mM HEPES at pH 7.4, 50 mM NaCl) and then initiated by addition of 100 µM ATP. The reactions were stopped after the noted period (30 min) then did immunoprecipitation with pS237A (custom antibody Thermo Scientific). Proteins were resolved by SDS-PAGE and immunoblot with Total S237A (custom antibody Thermo Scientific), with enhanced chemiluminescence (ECL Thermo Scientific)^35^.

### In vitro kinase and acetylation reaction method

In vitro kinase assays were by preincubating purified TAK1/TAB1 and the kinase substrate αTAT1 (0.5 μM each) in 1 mM MnCl_2_ for 5 min in buffer (20 mM HEPES at pH 7.4, 50 mM NaCl), then initiating the reaction by addition of 100 µM ATP. The reactions were stopped after 30 min. An aliquot of the kinase reaction was subsequently transferred to perform an in vitro acetylation reaction, carried out in 10 µl ADE buffer (40 mM Pipes pH 6.9, 0.8 mM EGTA, 0.5 mM MgSO_4_, 1 mM dithiothreitol) with purified bovine brain tubulin (1 µg; Cytoskeleton), phosphorylated αTAT1-WT and αTAT1-Ser237A, and 8 µM acetyl coenzyme A (Sigma). Reaction mixtures were incubated at 37 °C for 30 min and stopped by addition of SDS loading buffer. Proteins were resolved by SDS-PAGE and immunoblot for α-tubulin acetylation.

### Statistics

Statistical analysis was performed using both student t test and one-way analysis of variance (ANOVA). Data are presented as mean ± SEM. For crystal violet proliferation, data analysis was performed using Sigma plot, two-way ANOVA analysis/Bonferroni post-hoc analysis.

### Data availability

The authors confim that all data supporting the findings of this study are available within the paper and supplementary data.

## Electronic supplementary material

Supplementary Information
