# Supplementary material for: TAK1 activation of alpha-TAT1 and microtubule hyperacetylation control AKT signaling and cell growth
Source: Nat Commun. 2018 Apr 27;9:1696. doi: 10.1038/s41467-018-04121-y (PMC5923212; doi:10.1038/s41467-018-04121-y)

Supplementary Information

TAK1 activation of alpha-TAT1 and microtubule hyperacetylation control AKT signaling and cell growth

By Shah et al.

**
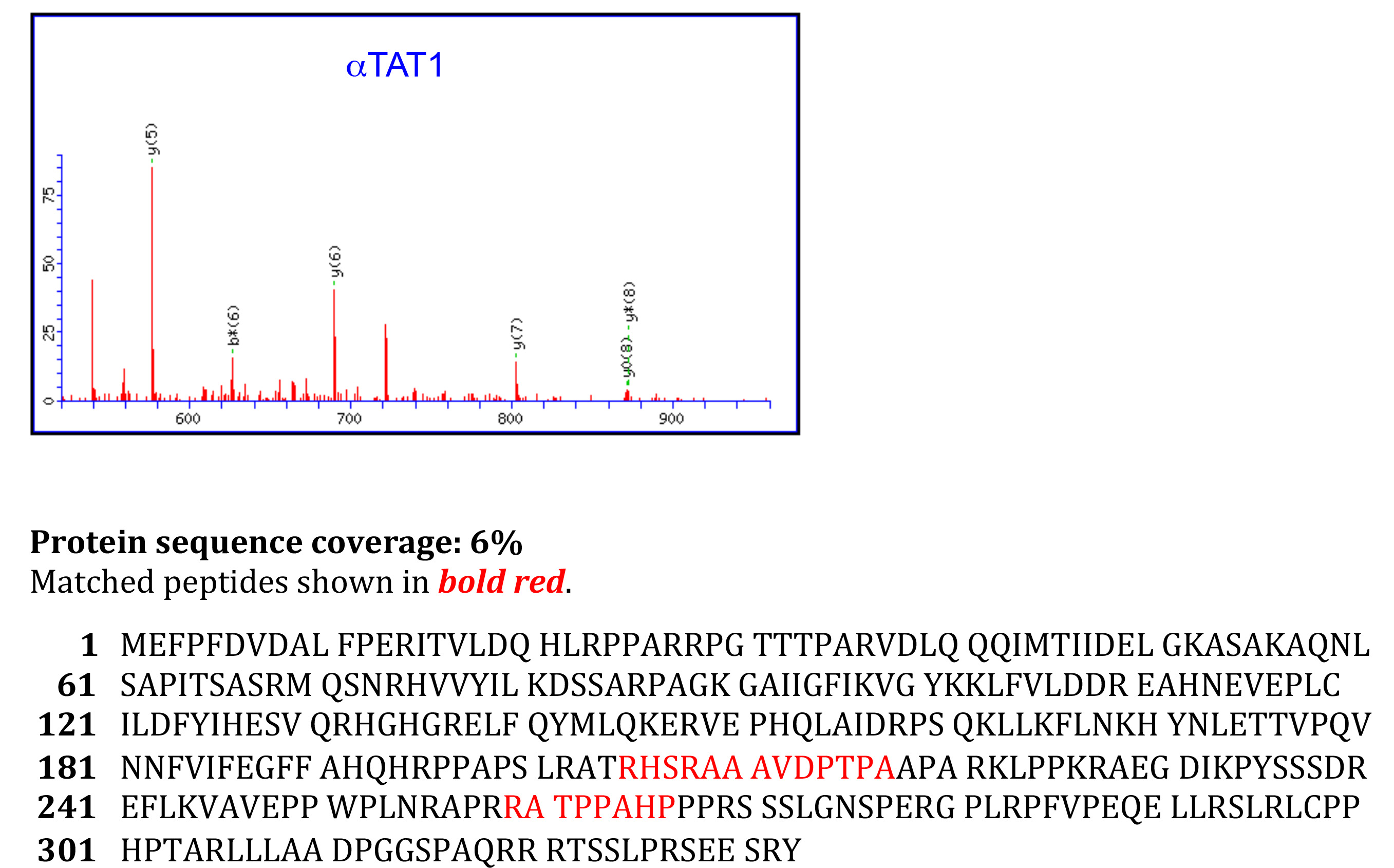
**

**Supplementary Figure 1. Identification of αTAT1 as a new TAK1 binding partner through mass spectrometry and proteomics analysis, Related to Figure 1.** Mass spectrometry peptide fragment identification of **α**TAT1. Immunoprecipitation of Flag-tagged TAK1 in COS-7 cells was subjected to nano LC-MS/MS and proteomics analyses. The mass spectrometry chromatogram peaks and internal sequence of the protein that matched to peptide fragments for **α**TAT1 are shown in red.


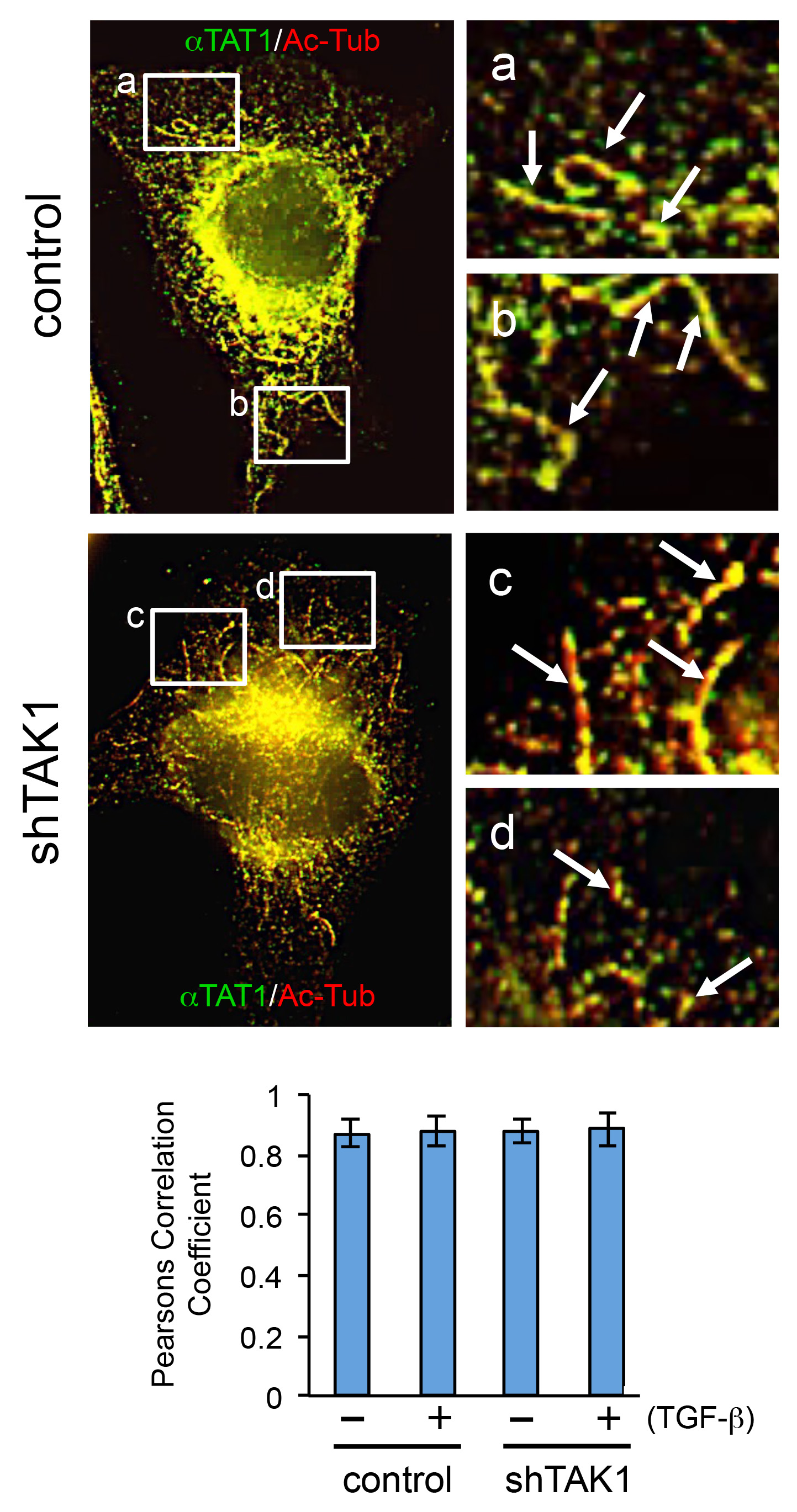


**Supplementary Figure 2. αTAT1 localization to the MTs is not altered by TAK1, Related to Figure 5.** HeLa scrambled (control) and HeLa shTAK1 cells were treated with TGF-β (200pM) for 30 mins prior to fixing and imaged for endogenous αTAT1 and acetyl tubulin. ROI a and b in control panel and c and d in shTAK1 panels are used to demonstrate the co-localization of αTAT1 and acetyl tubulin as indicated by white arrows. Pearsons correlation coefficient is calculated using Image J plugin JACoP. Quantification was based on data collected from 20 cells per condition and at least 2 ROI per cell. The images are representative of 3 independent experiments.


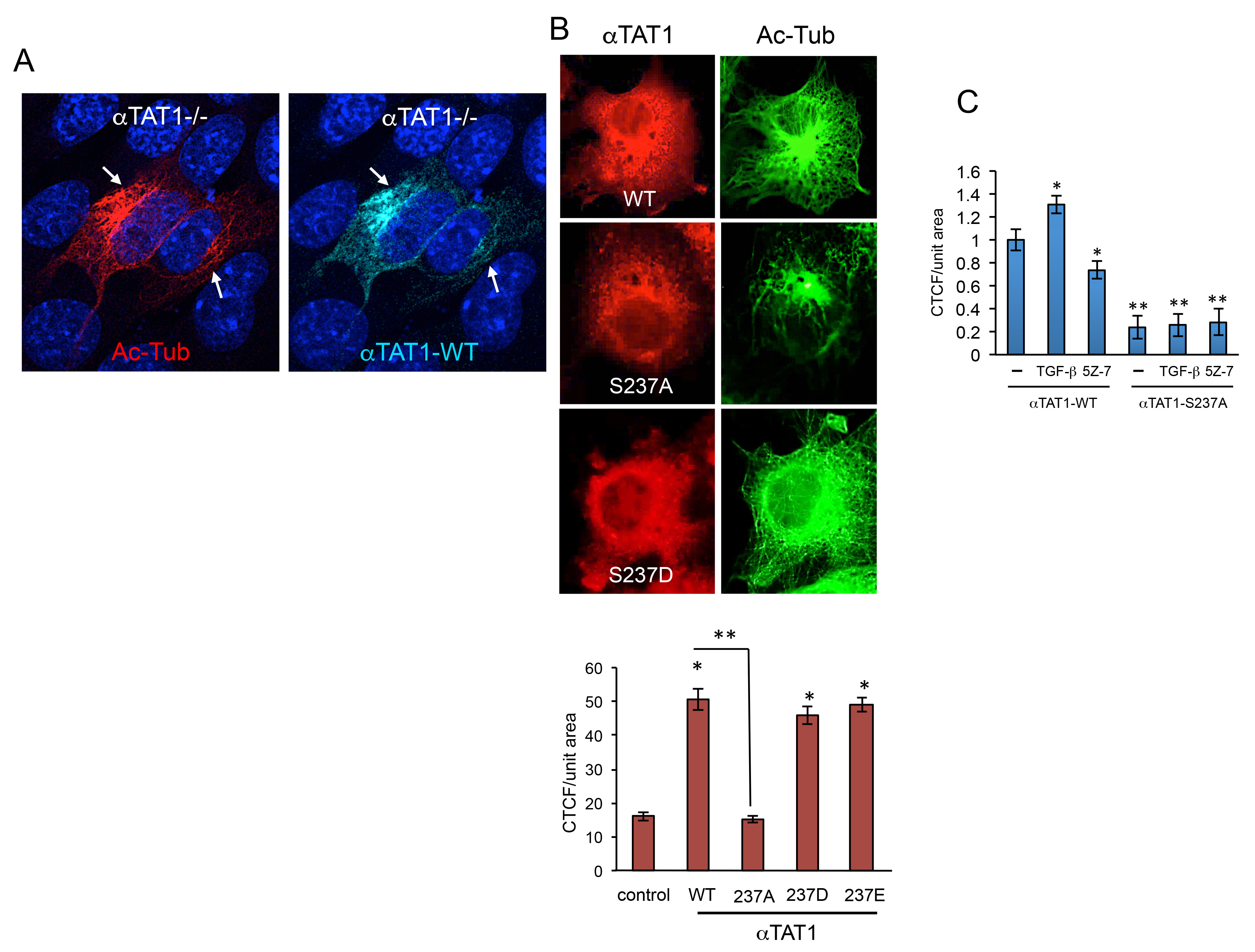


**Supplementary Figure 3. αTAT1 Ser237 phosphorylation regulates catalytic activity, Related to Figure 5.** (A) Immunofluorescence staining of acetyl tubulin (left) and αHA (right) representing acetyl tubulin levels in response to ectopic expression of HA-αTAT1 WT in αTAT1-/- MEFs. (B) COS-7 cells expressing HA-αTAT1 WT, HA-αTAT1 S237A, HA-αTAT1 S237D or HA-αTAT1 S237E were fixed and imaged for immunofluorescence staining of αHA (left) and acetyl tubulin (right). CTCF per unit area was calculated using Image J. Representative images are from 3 independent experiments. 25 cells per condition were analyzed from each experiment. Error bars represent SEM and type 2 t test analysis show relative to control: * p < 0.05, relative to αTAT1 WT ** p < 0.001. (C) COS7 cells expression HA-αTAT1 WT and HA-αTAT1 S237A were treated with TGF-β (200 pM) or 5Z-7-oxozeaenol (20 μM) for 30 min prior to fixing and imaged for immunofluorescence staining of acetyl tubulin and αHA. CTCF per unit area was calculated using Image J. 25 cells per condition were analyzed from 3 independent experiments. Error bars represent SEM and type 2 t-test analysis show relative to αTAT1 WT control: *p < 0.05, ** p < 0.001


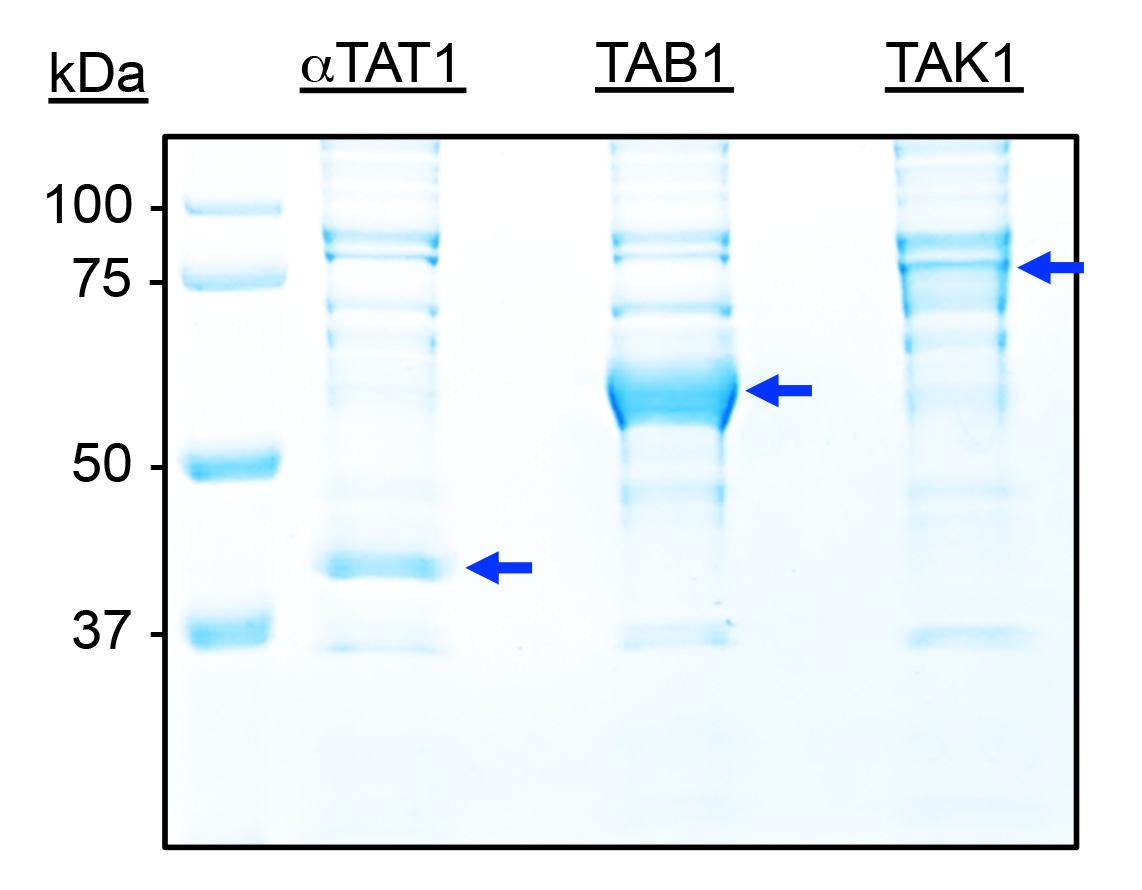


**Supplementary Figure 4. Coomassie staining of recombinant purified αTAT1, TAK1 and TAB1, Related to Figure 1 and 5.**


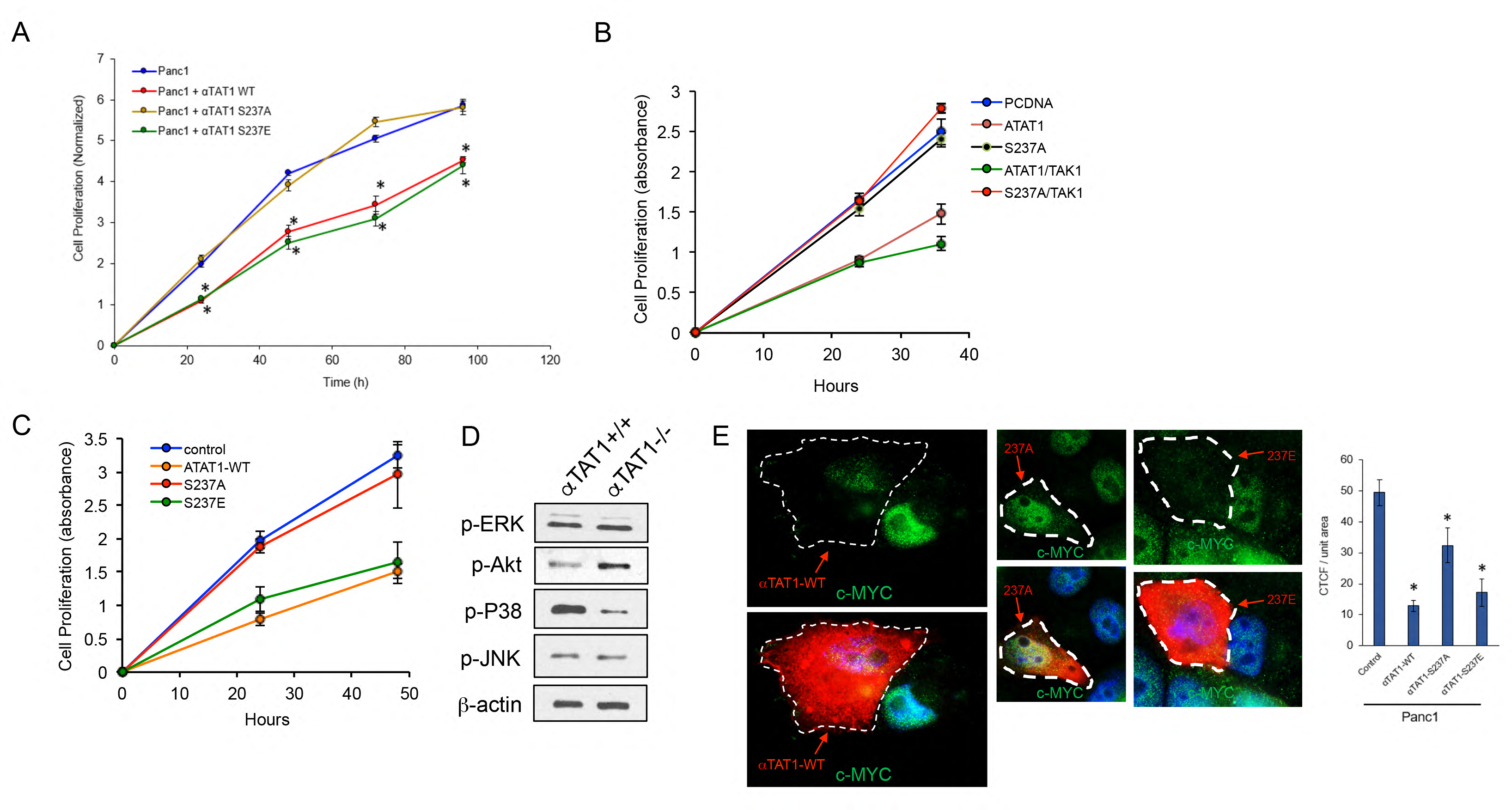


**Supplementary Figure 5. Regulation of cell proliferation by TAK1 phosphorylation of αTAT1, Related to Figure 6.** (A) Crystal Violet growth assay of Panc1 cells expressing HAαTAT1 WT, HA-αTAT1 S237A or HA-αTAT1 S237E over the period of 24, 48, 72 and 96 h. Data was analyzed by two-way ANOVA with Bonferroni *post-hoc* test: *p <0.05 (B) Crystal Violet growth assay of Hela cells expressing HA-αTAT1 WT, HA-αTAT1 S237A, or each co-expressed with TAK1 over the period of 24 and 36 h. Presented data is average of 3 independent experiments. (C) Crystal Violet growth assay of HeLa expressing HA-αTAT1 WT, HA-αTAT1 S237A or S237E over 24 and 36 h. Presented data is average of 3 independent experiments. (D) Representative western blots of p-ERK, p-Akt, p-P38, p-JNK and β-actin loading control. (E) Panc1 cells expressing HA-αTAT1 WT, HA-αTAT1 S237A or HA-αTAT1 S237E were fixed and imaged for αHA (red) and c-MYC (green) immunofluorescence co-staining. CTCF per unit area was quantified using Image J, at least 20 cells were quantified per condition. Error bars represent SEM and type 2 t-test analysis show relative to control: * p < 0.05.


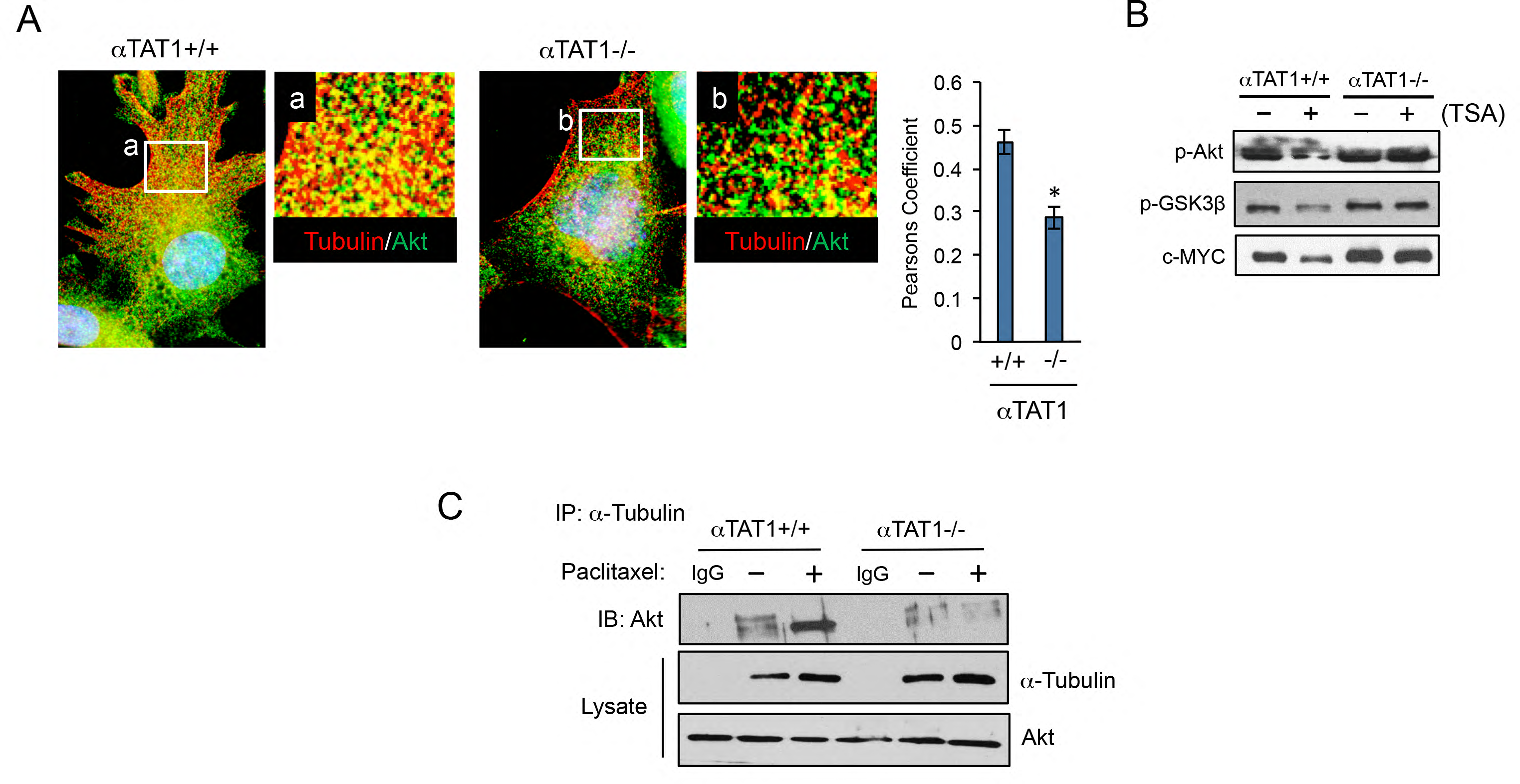


**Supplementary Figure 6. Related to Figure 6.** (A) Immunofluorescence images demonstrating localization of total tubulin (red) and total Akt (green) in αTAT1+/+ and αTAT1-/- MEFs. Pearsons correlation coefficient was calculated using JACoP, 30 cells per condition and 3 ROIs per cell were quantified. Error bars represent SEM and type 2 t-test analysis show relative to αTAT1+/+: * p < 0.05. (B) αTAT1+/+ and αTAT1-/- MEFs were treated with TSA (100 nM; 30 mins) prior to western blot analyses of the lysates for levels of pAKT S473, pGSK3β and c-MYC. (C) Total tubulin in αTAT1+/+ and αTAT1-/- MEFs was immunoprecipitated using anti-tubulin antibody (YOL1/34) and immunoblotted for total Akt levels.


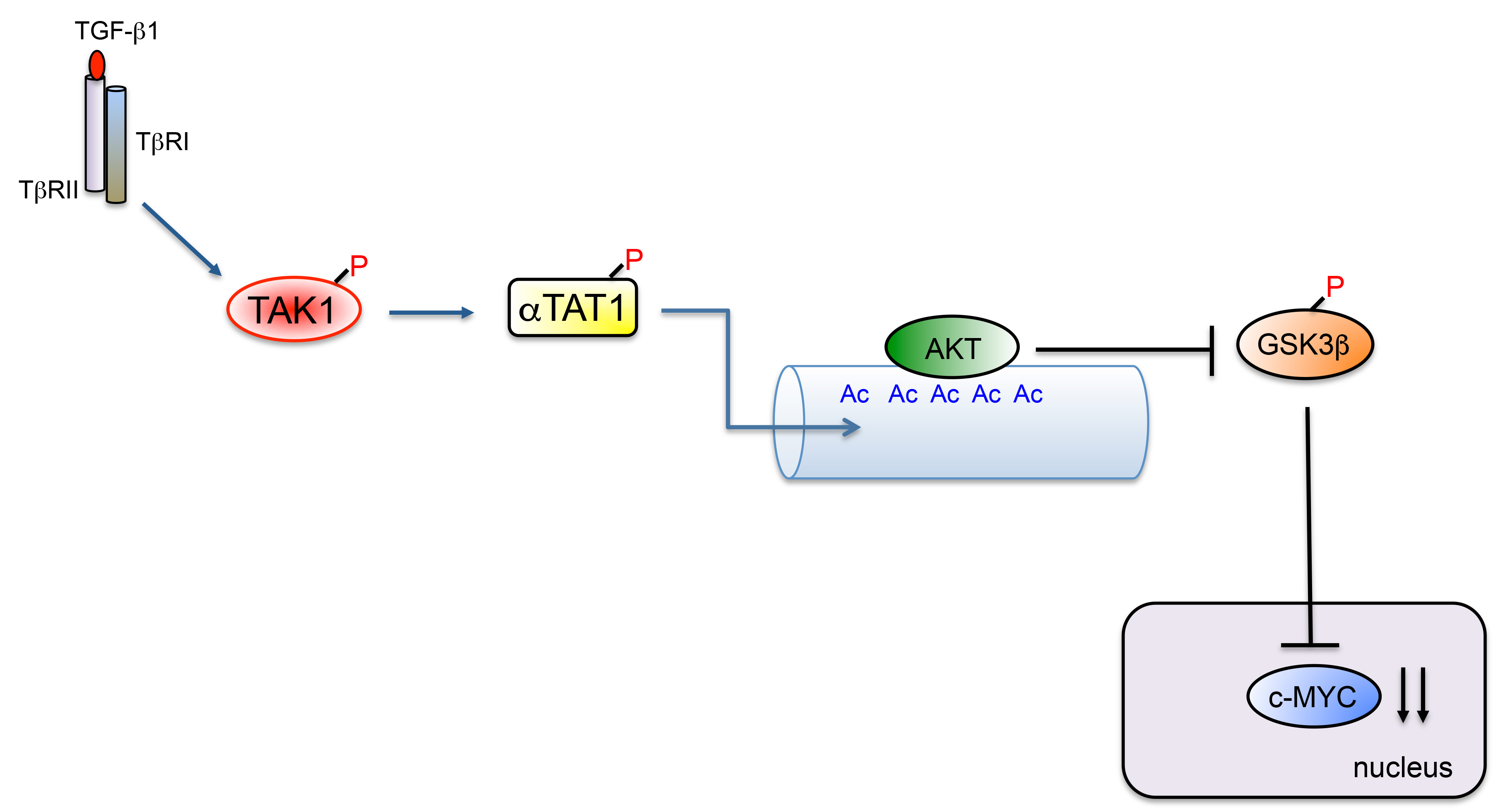


**Supplementary Figure 7. Working model of TAK1-induced MT acetylation and inhibition of cell proliferation.**

Supplementary Figure 8: Uncropped Western Blot Images Related to Figures 1-7


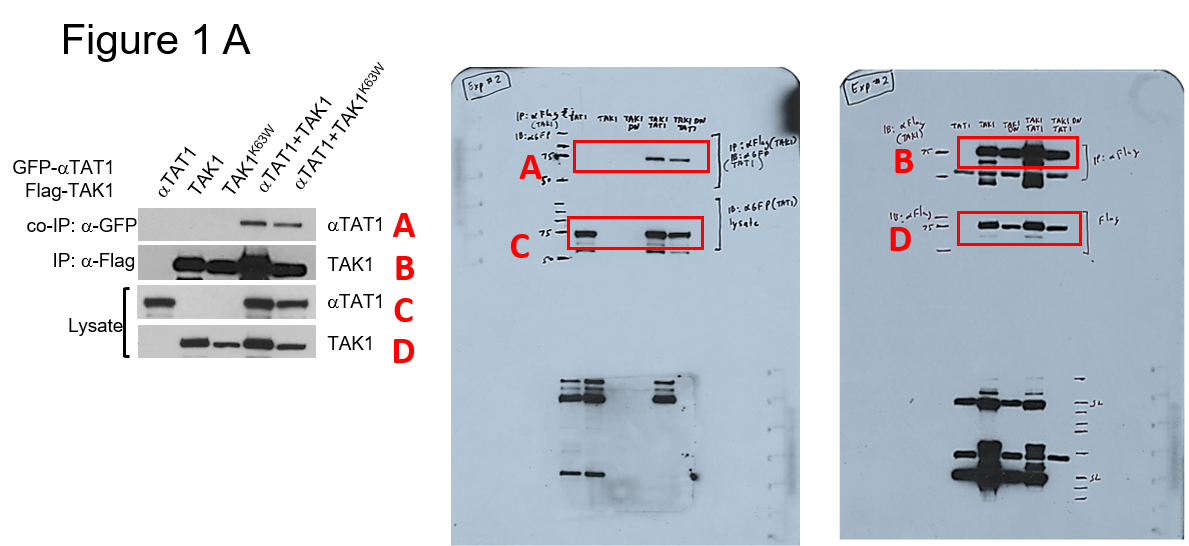

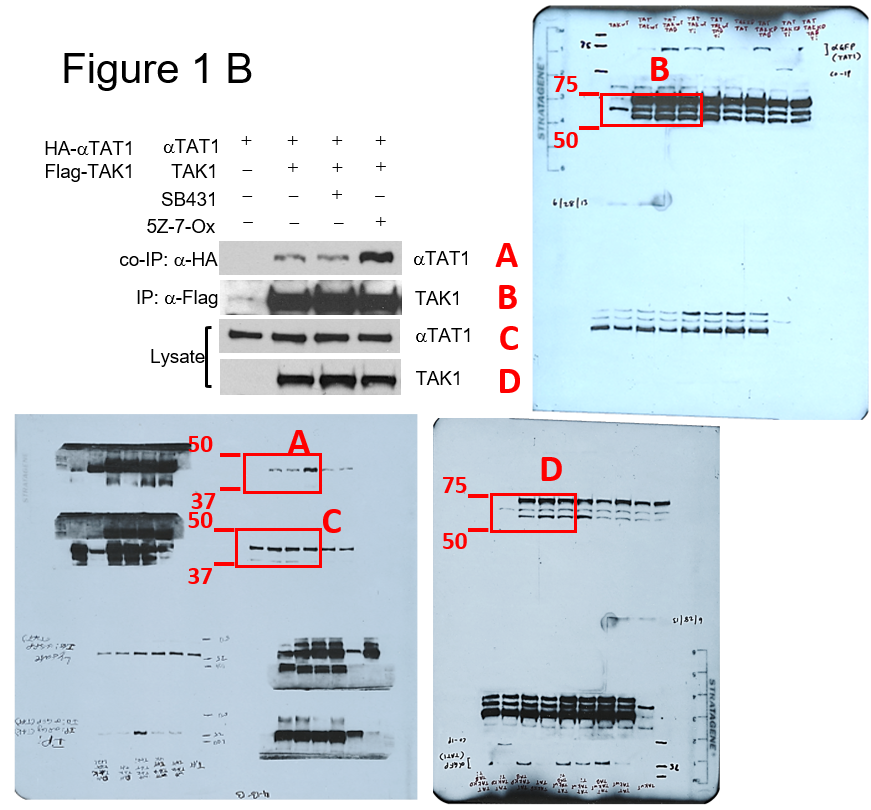


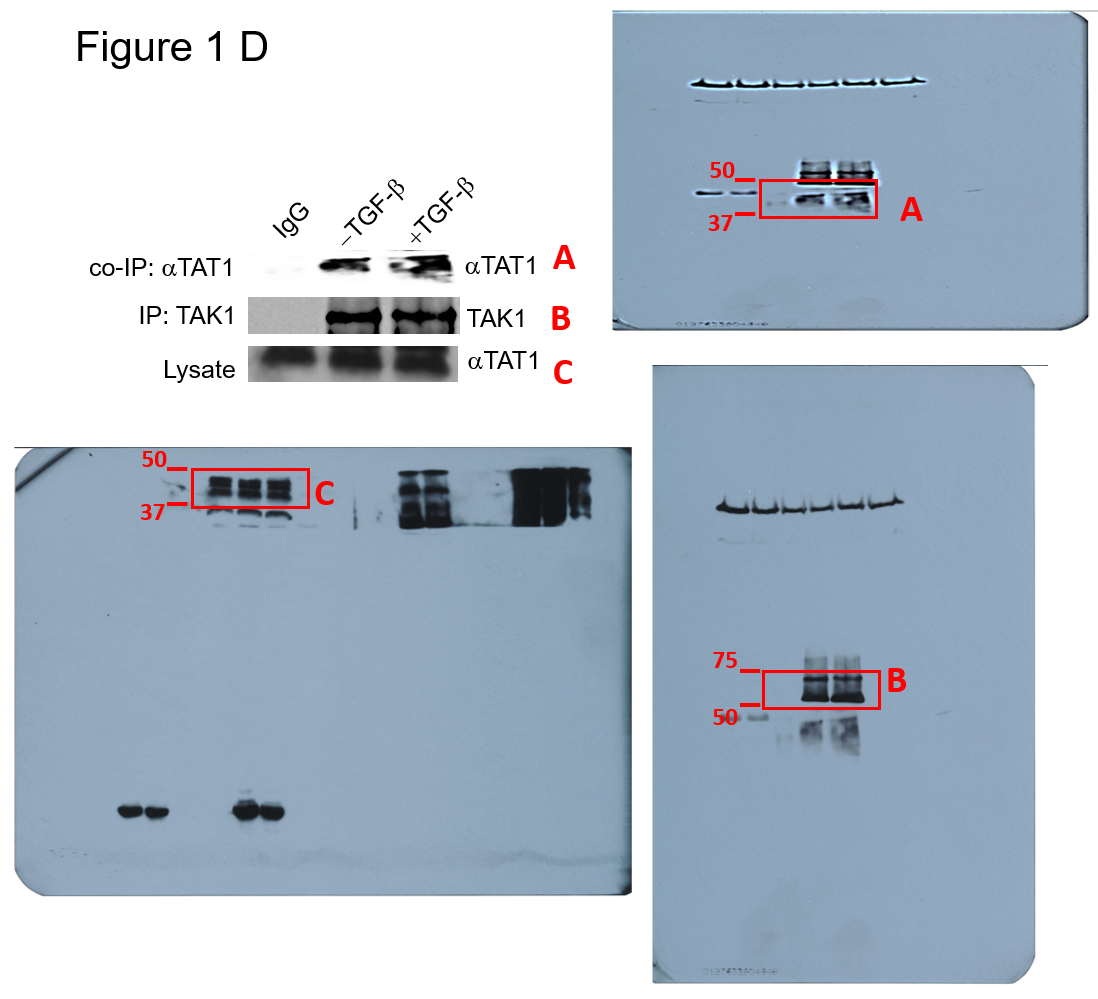


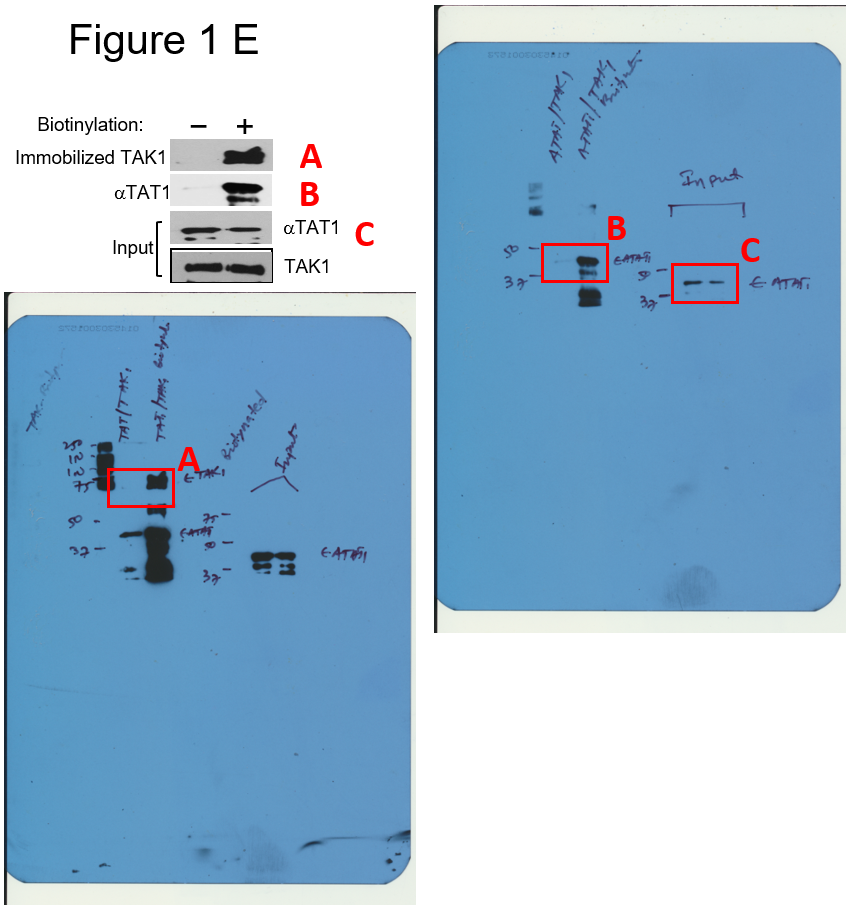


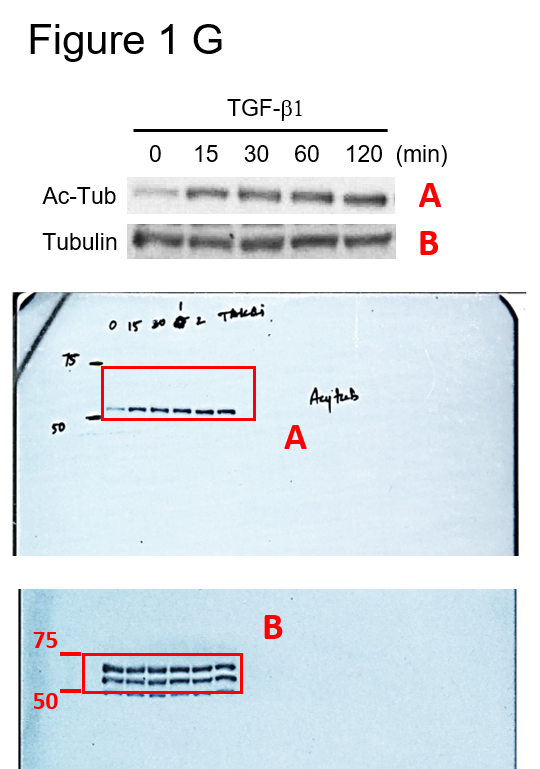


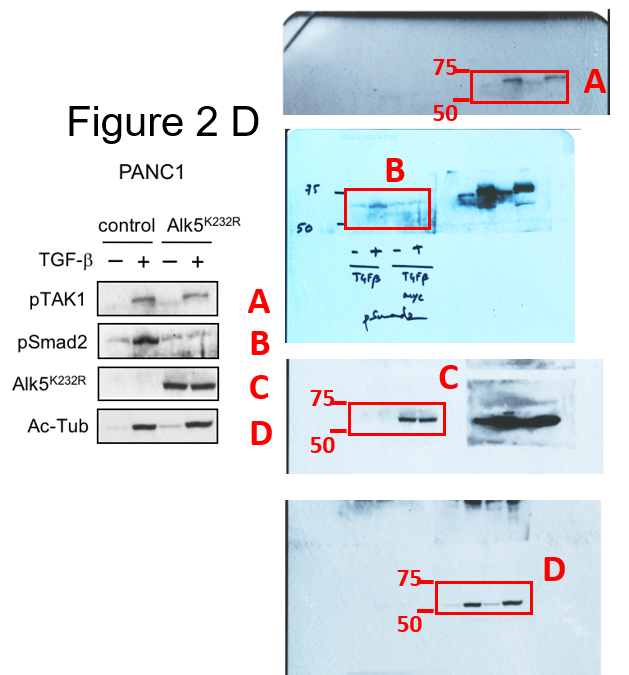


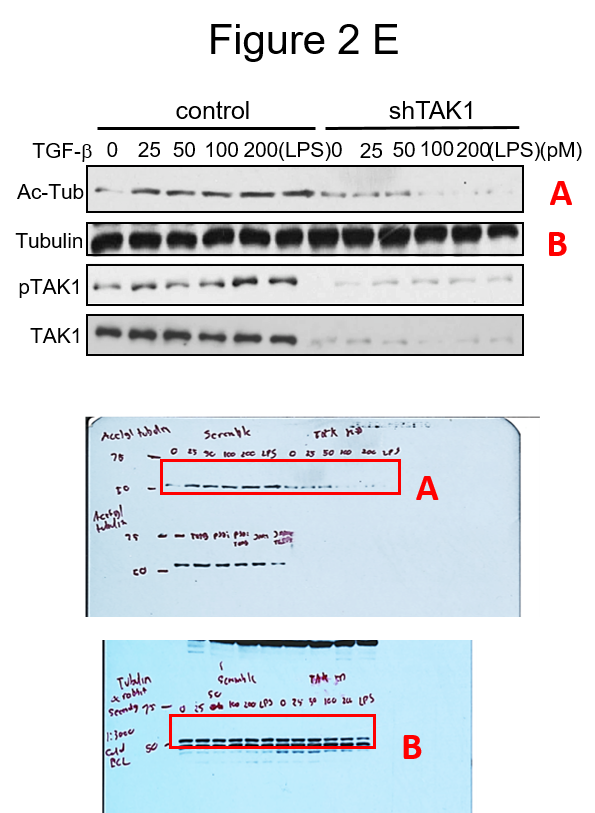


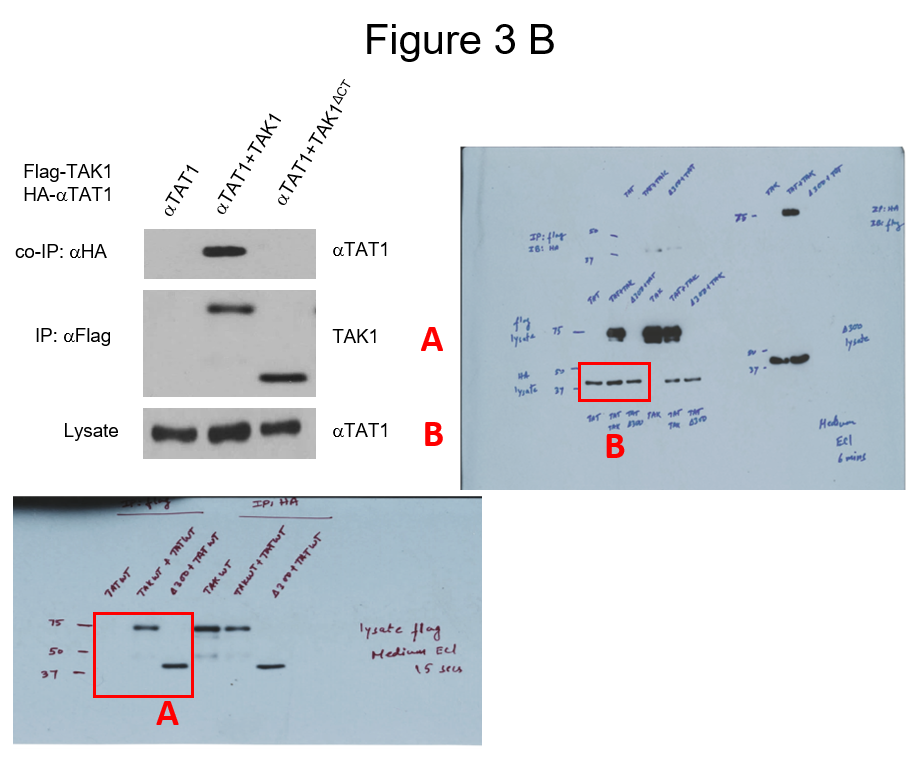


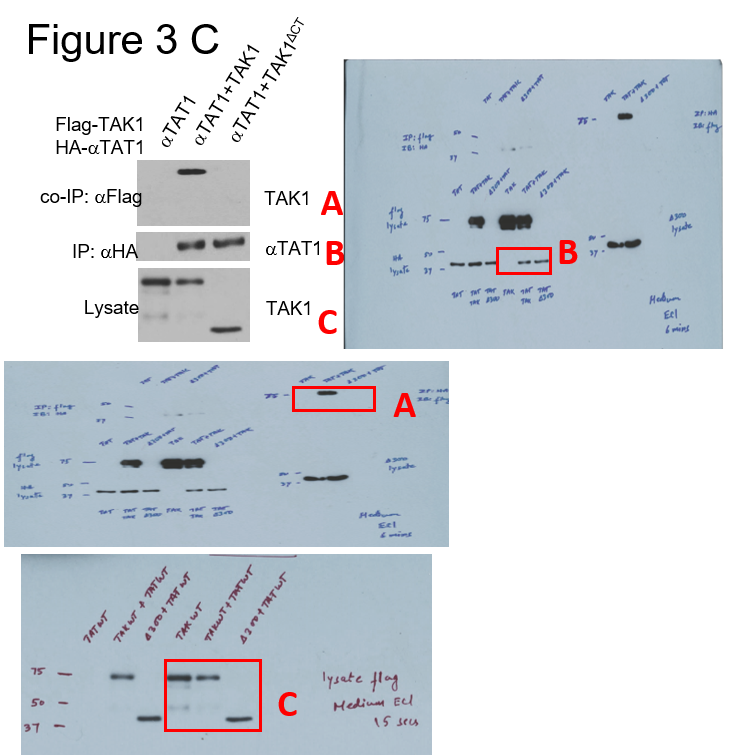


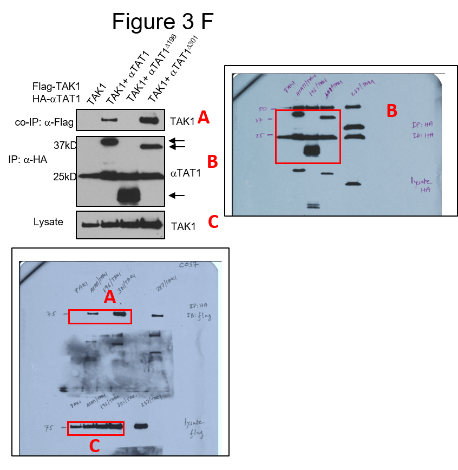


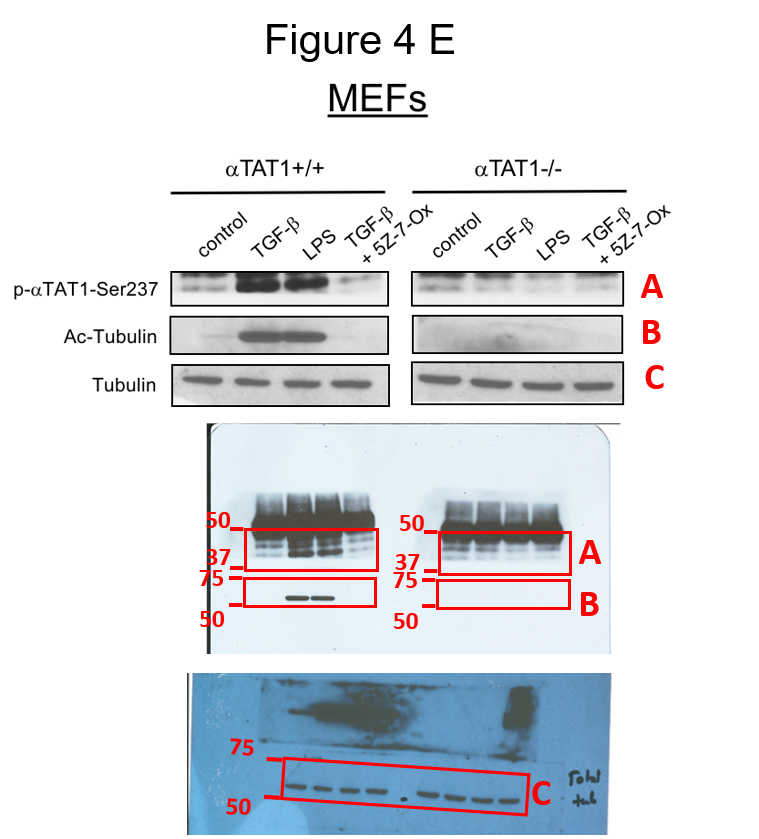


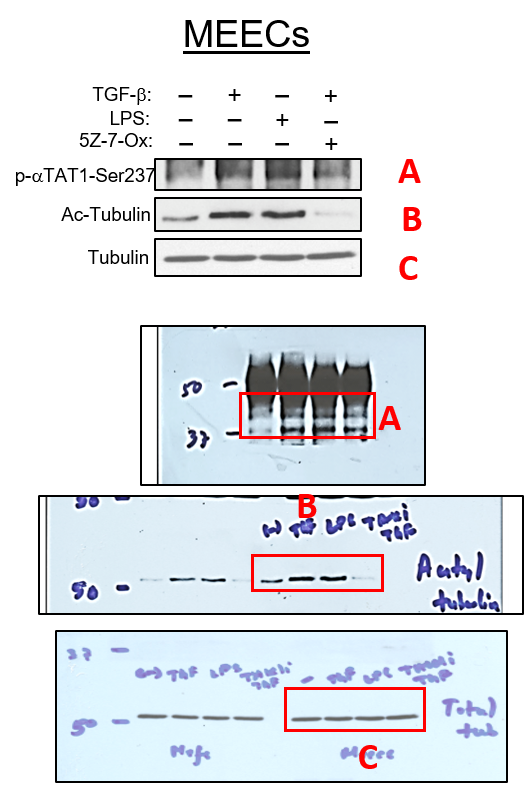


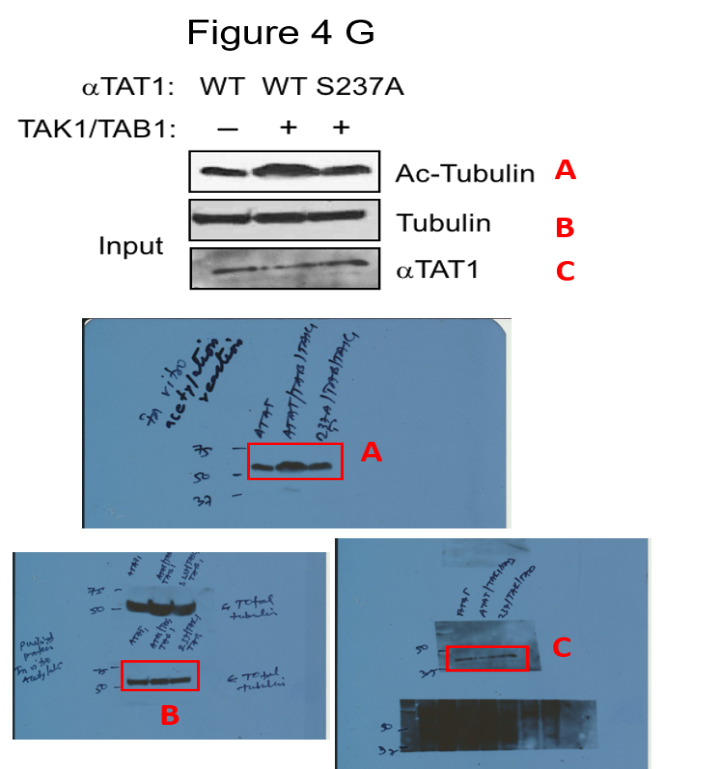

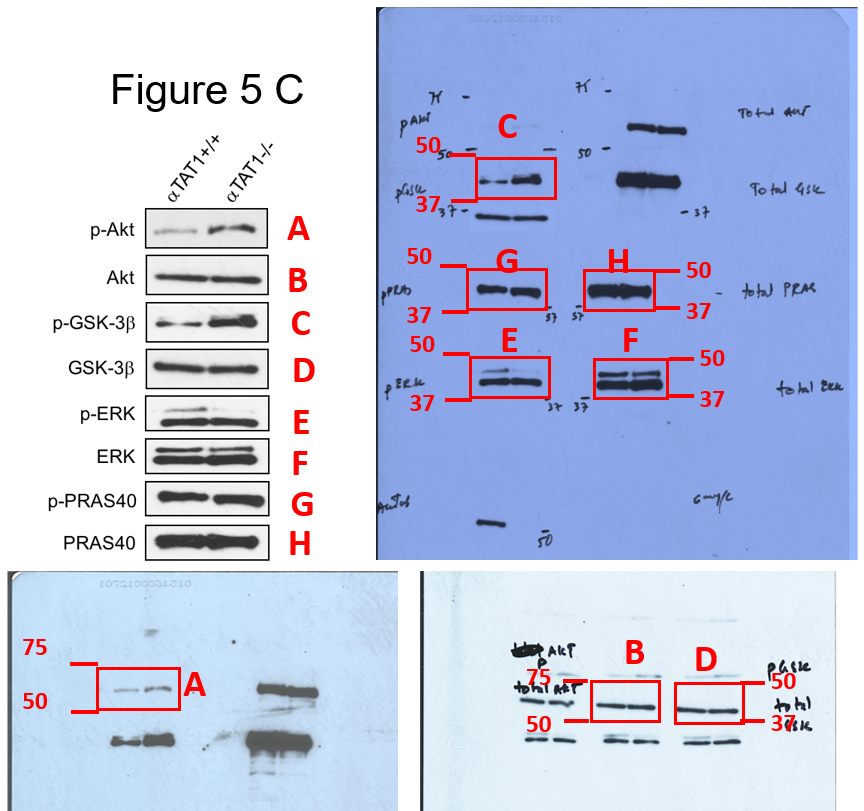


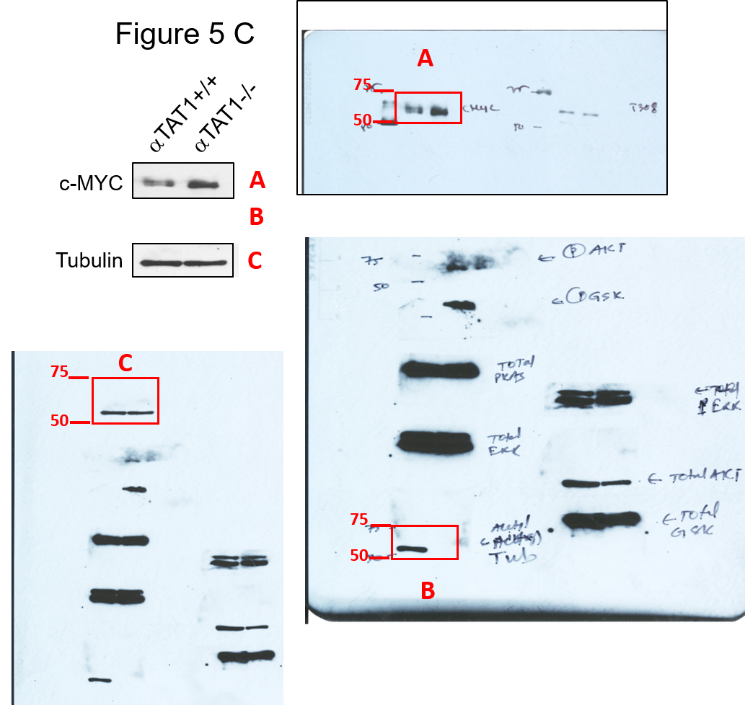


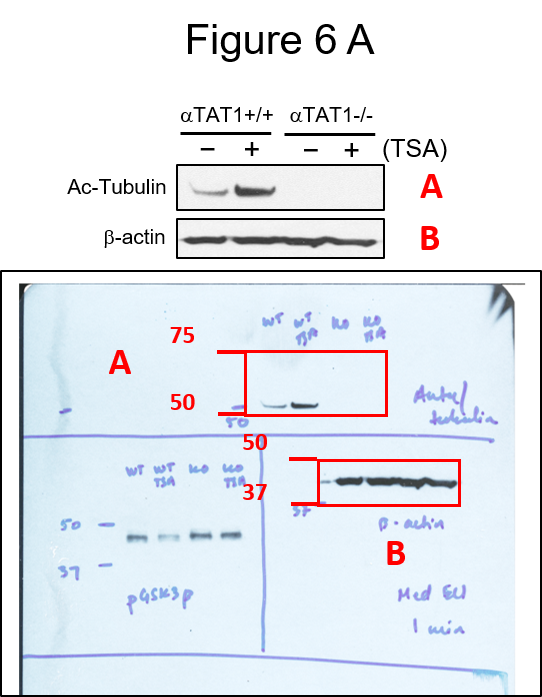


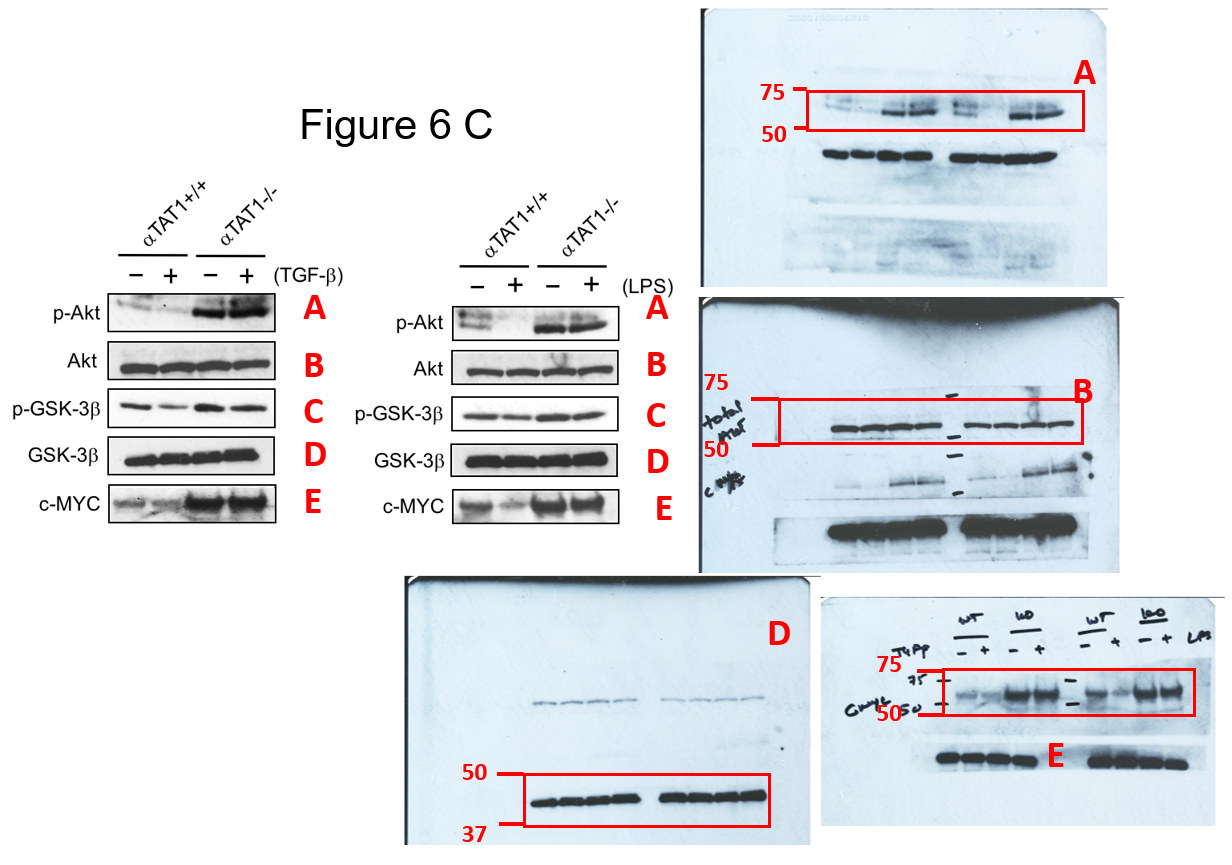


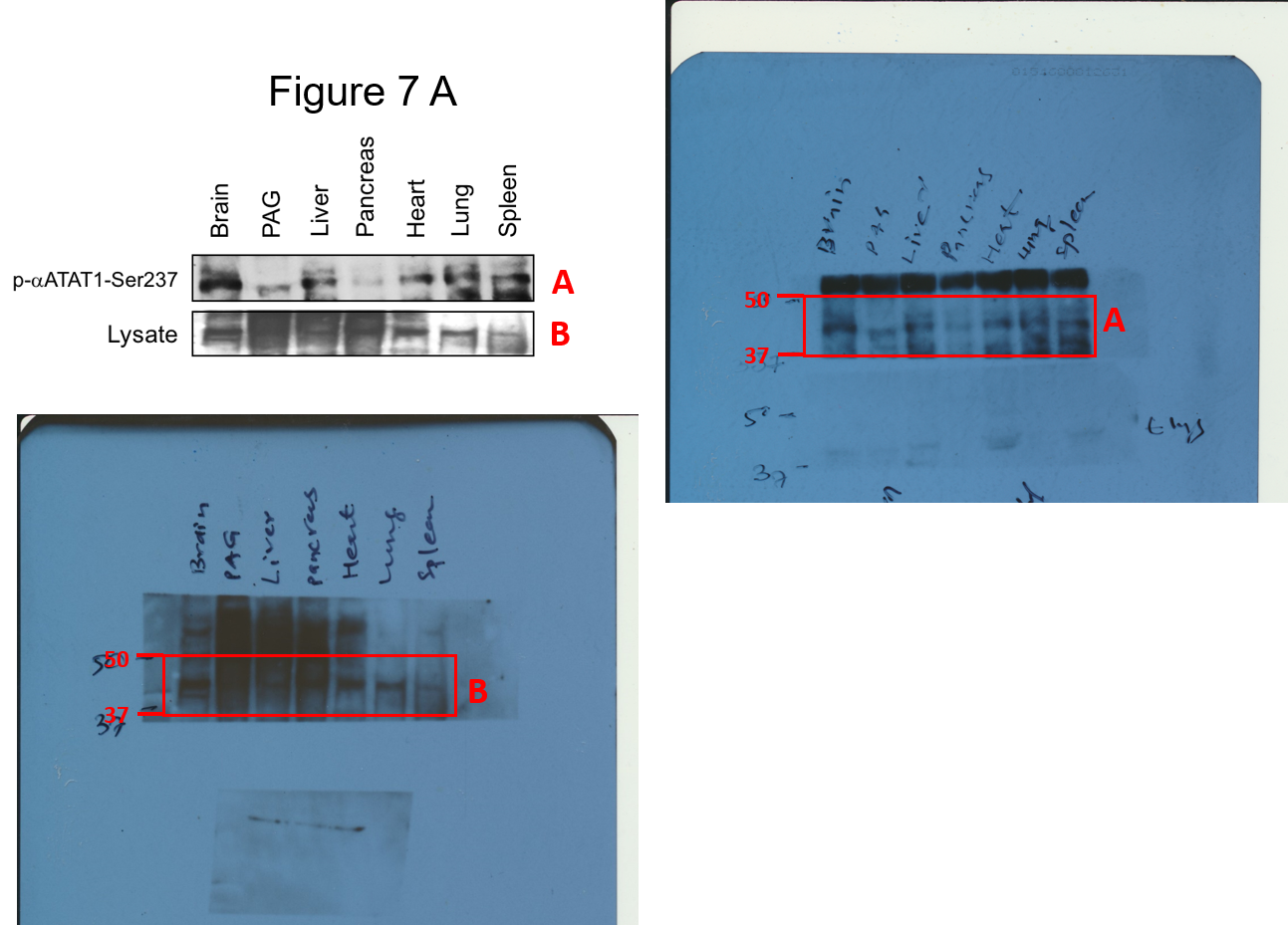


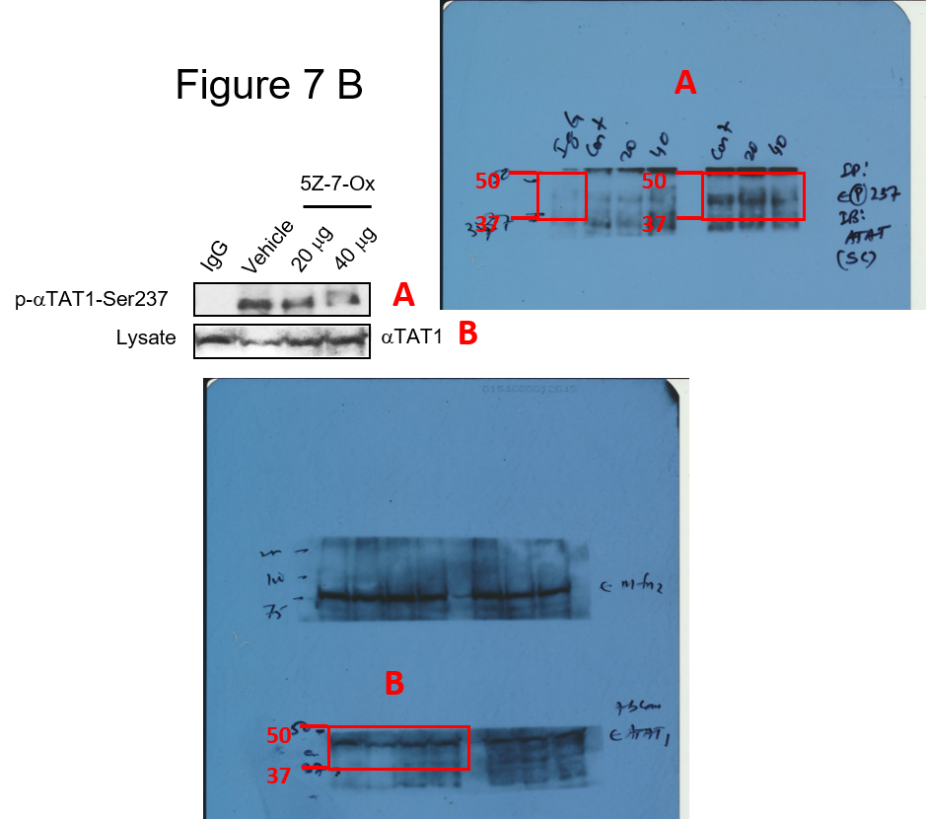


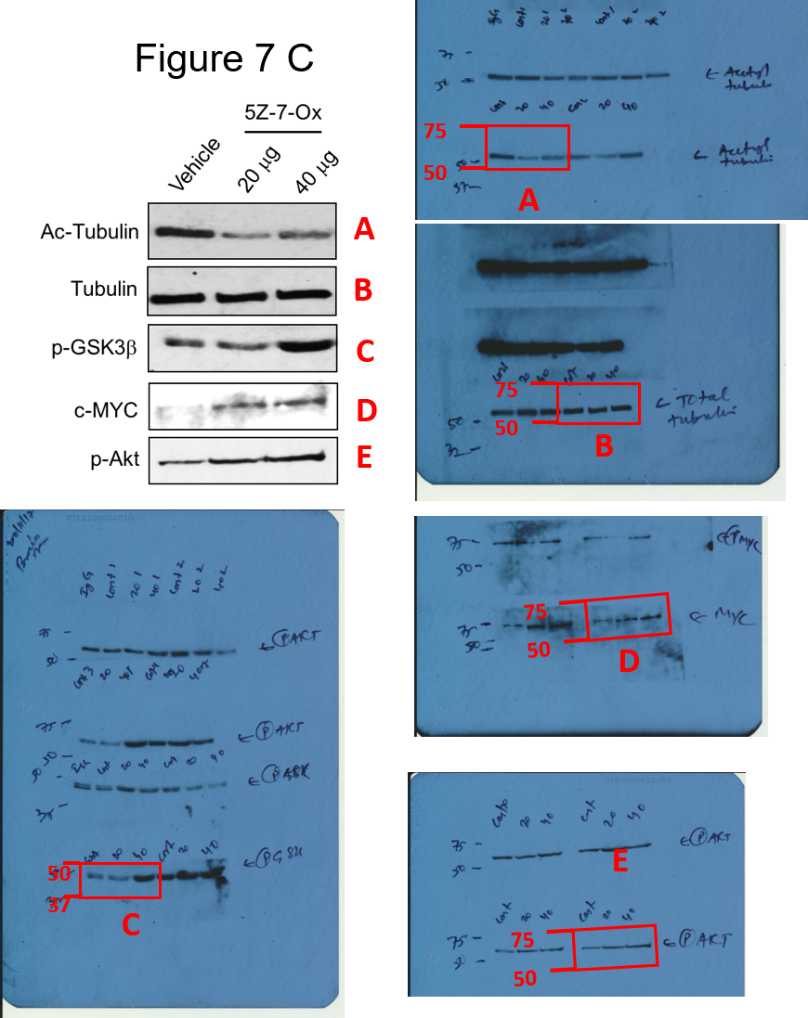

Supplement: Supplementary file 1 — Supplementary Information [file 41467_2018_4121_MOESM1_ESM.doc]
